# Endothelial-mesenchymal transition induced by metastatic 4T1 breast cancer cells in pulmonary endothelium in aged mice

**DOI:** 10.3389/fmolb.2022.1050112

**Published:** 2022-11-24

**Authors:** Marta Smeda, Agnieszka Jasztal, Ebrahim H Maleki, Anna Bar, Magdalena Sternak, Grzegorz Kwiatkowski, Joanna Suraj-Prażmowska, Bartosz Proniewski, Anna Kieronska-Rudek, Kamila Wojnar-Lason, Klaudia Skrzypek, Marcin Majka, Karolina Chrabaszcz, Kamilla Malek, Stefan Chlopicki

**Affiliations:** ^1^ Jagiellonian Centre for Experimental Therapeutics (JCET), Jagiellonian University, Krakow, Poland; ^2^ Department of Pharmacology, Jagiellonian University Medical College, Krakow, Poland; ^3^ Department of Transplantation, Faculty of Medicine, Institute of Pediatrics, Jagiellonian University Medical College, Krakow, Poland; ^4^ Faculty of Chemistry, Jagiellonian University in Krakow, Krakow, Poland; ^5^ Department of Experimental Physics of Complex Systems, Institute of Nuclear Physics, Polish Academy of Sciences, Krakow, Poland

**Keywords:** endothelial-mesenchymal transition, ageing, age-related endothelial dysfunction, breast cancer metastasis, Raman spectroscopy

## Abstract

Ageing is a major risk factor for cancer metastasis but the underlying mechanisms remain unclear. Here, we characterised ageing effects on cancer-induced endothelial-mesenchymal transition (EndMT) in the pulmonary circulation of female BALB/c mice in a metastatic 4T1 breast cancer model. The effect of intravenously injected 4T1 cells on pulmonary endothelium, pulmonary metastasis, lung tissue architecture, and systemic endothelium was compared between 40-week-old and 20-week-old mice. The 40-week-old mice showed features of ongoing EndMT in their lungs before 4T1 breast cancer cell injection. Moreover, they had preexisting endothelial dysfunction in the aorta detected by *in vivo* magnetic resonance imaging (MRI) compared to 20-week-old mice. The injection of 4T1 breast cancer cells into 40-week-old mice resulted in rapid EndMT progression in their lungs. In contrast, injection of 4T1 breast cancer cells into 20-week-old mice resulted in initiation and less pronounced EndMT progression. Although the number of metastases did not differ significantly between 20-week-old and 40-week-old mice, the lungs of older mice displayed altered lung tissue architecture and biochemical content, reflected in higher Amide II/Amide I ratio, higher fibronectin levels, and hypoxia-inducible factor 1 subunit alpha (HIF1α) levels as well as lower nitric oxide (NO) production. Our results indicate that age-dependent pre-existing endothelial dysfunction in the pulmonary endothelium of 40-week-old mice predisposed them to rapid EndMT progression in the presence of circulating 4T1 breast cancer cells what might contribute to a more severe metastatic breast cancer phenotype in these ageing mice compared to younger mice.

## 1 Introduction

Healthy endothelium determines cardiovascular homeostasis, and pulmonary endothelium is considered the orchestral conductor of respiratory diseases (Huertas et al., 2018). Although ageing is not itself a disease, it is associated with the progressive impairment of endothelial function that can be accelerated by diverse diseases (Seals et al., 2014), such as cancer (Toya et al., 2020), enhancing endothelial dysfunction progression by various mechanisms (Smeda et al., 2020a). Endothelial dysfunction in primary tumours, systemic circulation, and metastatic organs contributes significantly to malignant disease course and its terminal outcome (Smeda et al., 2020a). The impact of cancer-driven endothelial dysfunction is particularly evident in aged individuals (Mehta et al., 2018). Metastatic breast cancer is typically diagnosed in older women (Fusco et al., 2018), and its mortality rates increase with patient age (Derks et al., 2018). Surprisingly, clinical data show that while older females often succumb to malignant disease, they also often die from cancer or cancer treatment-related cardiovascular complications (Patnaik et al., 2011; Bradshaw et al., 2016; Stoltzfus et al., 2020). Rapid deterioration of endothelial function contributes significantly to mortality rates in older breast cancer patients, caused either by the progression (Buczek et al., 2018; Smeda et al., 2018) or treatment (Scott et al., 2018) of malignant disease, which may both have detrimental effects on the endothelium (Wojcik et al., 2015; Pacholczak et al., 2018), triggering rapid progression of cardiovascular diseases (Wojcik et al., 2015). Furthermore, cardiovascular diseases underlie most late comorbidities in cancer survivors (Daher et al., 2012). Therefore, advanced breast cancer patient age is a negative prognostic factor for outcome of the malignancy (Dhingra and Vasan, 2012) that also contributes to cardiovascular mortality in older cancer survivors. Consequently, a better understanding of the mechanisms responsible for increased age-related mortality in breast cancer patients is urgently needed.

One phenotypic change associated with endothelial dysfunction is the mesenchymal transformation of endothelial cells (EndMT). This endothelial phenotype change affects cancer growth and metastasis and may enable cancer cells to resist anti-cancer therapy (Platel et al., 2019). During EndMT, endothelial cells progressively lose the expression of endothelium-specific genes and begin to express mesenchymal markers (Gasparics et al., 2016). This transformation may give rise to cancer-associated fibroblasts and increased cancer metastasis (Gasparics et al., 2016). Indeed, we have previously shown that EndMT is an important component of the pre-metastatic niche in murine lungs in an orthotopic metastatic breast cancer model (Smeda et al., 2018). The pulmonary EndMT was characterised by lower levels of the endothelium-specific proteins vascular endothelial cadherin (VE-CAD), cluster of differentiation 31 (CD31), von Willebrand factor (vWF), vascular endothelial growth factor receptor 2 (VEGFR2), and the endothelium-specific isoform of nitric oxide (NO) synthase (eNOS), resulting in lower NO production. Impaired eNOS-derived NO bioavailability could reflect lower eNOS expression and activity or accelerated NO degradation, increased reactive oxygen species production, or altered NO production dynamics (Brandes et al., 2005; Toda, 2012). Altered endothelial capacity to release NO has multiple consequences, including changes in vascular tone, endothelial permeability, vascular smooth muscle cell (VSMC) proliferation, neointimal hyperplasia (Garg and Hassid A, 1989; Tsihlis et al., 2011), and ultimately EndMT (O’Riordan et al., 2007; Vanchin et al., 2019). NO deficiency also greatly impacts cancer cell endothelial adhesion (Stojak et al., 2018).

In this study, we tested the hypothesis that ageing affects the pulmonary endothelial response to intravenously (i.v.) injected 4T1 breast cancer cells in BALB/c mice. We show that ongoing EndMT in the lungs of 40-week-old mice predisposed them to rapid EndMT progression in the pulmonary endothelium in response to i.v. injected 4T1 breast cancer cells, potentially representing a significant determinant of the outcome of metastatic disease in the older mice.

## 2 Materials and methods

### 2.1 Animals

Female BALB/cJRj mice were obtained from Janvier Labs (Le Genest-Saint-Isle, France). These included 90 mice aged 20 weeks and 101 aged 40 weeks (retired breeders). Note that 20-week-old mice are considered young adults, while 40-week-old are considered middle-aged (Jackson Laboratory, 2021). The mice were housed five per cage in a temperature-controlled environment (22–25°C), 12-hour light/day cycle, and unlimited access to food (Zoolab; Krakow, Poland) and water throughout the experiment. Mouse welfare was monitored daily throughout the study. Euthanasia was performed by intraperitoneal (i.p.) injection of ketamine and xylazine at 100 and 10 mg kg^−1^ of body weight, respectively, at designated timepoints. All experimental procedures involving animals have been carried out according to the Polish Research Council’s Guide for the Care and Use of Laboratory Animals under the consent issued by the Second Local Ethical Committee on Animal Testing, Institute of Pharmacology in Krakow, Poland (Permit Nos: 61/2020, 167/2020, 228/2020, and 66/2021).

### 2.2 Cell culture

This study used the mouse mammary adenocarcinoma 4T1-luc2-tdTomato cell line that stably expresses the firefly luciferase and tdTomato fluorescent genes. This cell line was provided by Professor Joanna Wietrzyk of the Ludwik Hirszfeld Institute of Immunology and Experimental Therapy at the Polish Academy of Sciences from the fifth passage after resuscitation following the purchase of the parental cell line (American Type Culture Collection (ATCC) CRL-2539) from Caliper Life Sciences Inc. (Hopkinton, MA, United States). The 4T1 breast cancer cells were cultured as previously described (Smeda et al., 2020b). Prior to i.v. injection, the cells were detached using Accutase solution (Sigma-Aldrich, Poznan, Poland) and centrifuged at 300 × *g* at 4°C for 5 min. Next, they were stained with Cell Tracker™ Red CMTPX Dye (Invitrogen; Waltham, MA, United States) for 30 min at 37°C to enable pulmonary metastases quantification 2 days post injection, rinsed three times with Dulbecco’s phosphate-buffered saline (DPBS; Gibco; Waltham, MA, United States), and a 1:1 ratio of DPBS and Hank’s Balanced Salt Solution (HBSS; IIET; Wroclaw, Poland). Then, they were resuspended in HBSS at the required concentration and injected into the tail vein of female BALB/c mice (7.5 × 10^4^ cells in 100 μl of HBSS per mouse). Cell cultures were tested routinely for *Mycoplasma* contamination.

### 2.3 Pulmonary endothelium permeability

Pulmonary permeability was measured in healthy control and 4T1 breast cancer cell injected mice by i.v. injection of Evans blue dye solution as previously described (Smeda et al., 2018). After inducing anesthesia (100 mg kg^−1^ ketamine with 10 mg kg^−1^ xylazine, i.p.), mice were injected i.v. with a solution of Evans Blue (EB; 60 kDa) dye (Sigma Aldrich) at a dose of 4 ml kg^−1^ and left for 10 min to allow it to circulate. Then, the lungs were perfused with phosphate-buffered saline (PBS) for 15 min, isolated, dry-weighted and homogenised in 200 μl of 50% trichloroacetic acid (TCA) dissolved in distilled water. The homogenates were centrifuged at 12,000 rpm for 12 min at 4°C and the supernatant was diluted in a 1:3 volume with 95% ethanol prior to photospectrometric determination of EB concentration (Synergy 4; Bio-Tek; Winooski, VT, United States) by excitation at 590 nm, emission at 645 nm, and absorbance at 620 nm. Data were normalised to lung weight.

### 2.4 Measurement of nitric oxide production in the lungs

To measure pulmonary eNOS-dependent NO production, we used electron paramagnetic resonance (EPR) spin trapping with diethyldithiocarbamic acid sodium salt *ex vivo* with minor modifications as described previously (Bar et al., 2019a). Lungs were perfused with ice-cold PBS buffer and excised. Next, 30 mg of the sample was cut into pieces and preincubated with 10 μmol l^−1^ N6‐(1‐iminoethyl)‐lysine, hydrochloride in N-2-hydroxyethylpiperazine-N′-2-ethanesulfonic acid (Krebs‐HEPES) buffer for 30 min at 37°C. The addition of N6‐(1‐iminoethyl)‐lysine hydrochloride during the preincubation period enabled the direct measurement of NO produced by eNOS since it inhibits NO production by inducible NO synthase (iNOS). Separately, to prepare the spin trap, 3.6 mg of diethyldithiocarbamic acid sodium salt and 2.25 mg of ferrous sulfate heptahydrate (FeSO_4_ 7H_2_O) were dissolved under argon gas bubbling into two 10‐ml volumes of ice‐cold Krebs‐HEPES buffer and kept under gas flow on ice until used. After preincubation, the spin trap (125 μl of FeSO_4_ 7H_2_O and 125 μl of diethyldithiocarbamic acid sodium salt; final colloid concentration 285 μmol l^−1^) was added to each lung sample and incubated for 90 min at 37°C to detect basal NO release. Finally, lyophilised lung samples were weighed, suspended in fresh buffer, and frozen in liquid nitrogen in the middle of a 400‐μl Krebs‐HEPES buffer column and stored at −80°C until measurement. EPR spectra were obtained using an X‐band EPR spectrometer (EMX Plus; Bruker; Munich, Germany) equipped with a H102 rectangular resonator cavity. Signals were quantified by measuring the total amplitude of the Fe(II)‐diethyldithiocarbamate after baseline correction. The quantitative EPR-determined NO production results are expressed in arbitrary units per mg of tissue.

### 2.5 Magnetic resonance imaging-based assessment of endothelial function in systemic and pulmonary circulation *in vivo*

#### 2.5.1 Measurement of endothelium-dependent vasodilation in the aorta in response to acetylcholine

Endothelial function was assessed *in vivo* using an MRI-based method well-validated in our previous studies (Bar et al., 2016; Bar et al., 2019b; Bar et al., 2020) that involves quantifying of endothelium-dependent response to acetylcholine (ACh) administration. The endothelium-independent response induced by sodium nitroprusside (SNP) was also assessed for comparison. Response to ACh (Sigma-Aldrich: 50 μl, 16.6 mg kg^−1^, *i.p*.) or SNP (Sigma-Aldrich: 1 mg kg^−1^, *i.v*.) injection was analysed in the abdominal (AA) and thoracic (TA) aorta. Vasomotor responses were assessed by comparing two time-resolved three-dimensional (3D) images of the vessels prior to and 30 min after their administration. 3D images of the aorta were acquired using the cine IntraGate™ FLASH 3D sequence and reconstructed with the IntraGate 1.2.b.2 macro (Bruker). Analysis was performed using a 9.4T scanner (BioSpec 94/20 USR; Bruker, Germany). During MRI experiment, mice were anaesthetised using 1.5% isoflurane (Aerrane; Baxter Sp. z o. o.; Warszawa, Poland) in a 1:2 oxygen to air mixture and imaged in the supine position. Heart function (rhythm and electrocardiogram (ECG)), respiration, and body temperature (maintained at 37°C using circulating warm water) were monitored using a Monitoring and Gating System (SA Inc.: Stony Brook, NY, United States). Vasodilation was assessed using the ImageJ software 1.46r (National Institute of Health; Bethesda, MD, United States) and scripts written in Matlab (MathWorks; Natick, MA, United States) in the hyperstack of the AA (10 slices in diastole, from the renal arteries down) and TA (10 slices in diastole, from the celiac artery up). Percentage changes in the vessel volume after ACh or SNP administration were calculated. Imaging parameters included: repetition time (TR; 6.4 ms); echo time (TE; 1.4 ms); the field of view (FOV; 30 mm^3^ × 30 mm^3^ × 14 mm^3^); matrix size (256 × 256 × 35); flip angle (−30°), and accumulation number (NA; 15). Images were reconstructed into seven cardiac frames. The total scan time was about 12 min.

#### 2.5.2 Assessment of NO-dependent function in pulmonary endothelium based on MRI-based T1 mapping

Pharmacological eNOS inhibition with 100 mg kg^−1^ of N^G^‐nitro‐L‐arginine methyl ester (L-NAME) leads to microvascular fluid efflux that represents NO-dependent regulation of the endothelial barrier. These changes can be detected non-invasively *in vivo* using MRI to track tissue longitudinal relaxation time (T_1_) changes, an endogenous molecular marker of water-protein tissue content (Cui and Epstein, 2018). The longitudinal relaxation maps were recorded with a variable flip angle (VFA) approach (Wang et al., 1987) using a set of six radiofrequency (RF) excitation angles (2, 5, 8, 13, 20, and 50°) and echo-time (UTE) sequence (Alamidi et al., 2018) provided in ParaVision 6.0.1 (Bruker BioSpec; Ettlingen, Germany). The following parameters were used: FOV: 30 mm^3^ × 30 mm^2^; reconstruction matrix:128 × 128; echo/repetition time: 0.26/10 ms; number of projections per repetition: 402; number of averages: 16; receiver bandwidth: 200 kHz. The images were reconstructed using ParaVision 6.0.1 with the manufacturer’s provided routine for trajectory measurements. The T_1_ maps were recorded twice before and six times after *i.v.* injection of 100 mg/kg of L-NAME, with a time step of 6 min. The absolute change in tissue T_1_ is presented as a percentage change relative to the baseline (before L-NAME injection).

### 2.6 Assessment of systemic endothelial dysfunction biomarkers

The plasma concentration of a panel of endothelial dysfunction biomarkers was quantified using microLC/MS-MRM method as described previously (Walczak et al., 2015; Suraj et al., 2018; Suraj et al., 2019a; Suraj et al., 2019b; Smeda et al., 2022) and a UPLC Nexera system (Shimadzu; Kyoto, Japan) connected to a highly sensitive QTrap 5500 mass spectrometer (Sciex; Framingham, MA, United States). The panel included eighteen glycocalyx disruption biomarkers: syndecan-1 (SDC-1) and endocan (ESM-1); hemostasis: von Willebrand factor (vWF), tissue plasminogen activator (t-PA), plasminogen activator inhibitor 1 (PAI-1), and thrombin activatable fibrinolysis inhibitor (TAFI); endothelium inflammation: soluble vascular cell adhesion molecule 1 (sVCAM-1), soluble intercellular adhesion molecule 1 (sICAM-1), and soluble form of E-selectin (sE-sel); platelet activation: soluble form of P-selectin (sP-sel) and thrombospondin 1 (THBS-1); endothelium permeability: angiopoietin 1 (Ang-1), angiopoietin 2 (Ang-2), soluble form of FMS-like tyrosine kinase 1 (sFLT-1), and soluble form of Tie-2 receptor (sTie-2); other proteins and peptides related to endothelial function: adrenomedullin (ADM), adiponectin (ADN) and annexin V (ANXA5). A detailed description of the targeted analysis of a selected panel of proteins and one peptide was presented elsewhere (Suraj et al., 2018; Suraj et al., 2019a; Suraj et al., 2019b).

### 2.7 Quantification of pulmonary metastasis

The lungs of 4T1 breast cancer cell-injected mice were excised upon euthanasia two and 7 days after i.v. inoculation, washed with saline, fixed in formalin, paraffin-embedded, and cut into 5 μm slices. The number of pulmonary metastases 2 days after injection of 4T1 breast cancer cells was quantified based on their vivid and stable red fluorescence while the metastatic count on the seventh day was assessed based on classical haematoxylin and eosin (H&E) staining. The number of pulmonary metastases was normalised to the area of the lung cross-section.

### 2.8 Immunohistochemical staining

Paraffin-embedded lungs were cut into slices and mounted on slides. Endothelium-specific snail family transcriptional repressor (Snail) levels in the lungs were quantified as previously described (Smeda et al., 2018). For α-smooth muscle actin (α-SMA; ab124964, Abcam, Cambridge, United Kingdom) staining, 5 µm sections were deparaffinised and antigen retrieval was performed in citrate buffer according to standard protocol. Next, lung cross-sections were stained with the anti-αSMA antibody (1: 2500) overnight at 4°C. Then, the cross-sections were incubated with the biotinylated goat anti-rabbit secondary antibody (1: 600; 111-065-144; Jackson ImmunoResearch; West Grove, PA, United States) for 30 min. The avidin-biotin complex (ABC; PK-4000; Vector Labs; Newark, CA, United States) was then applied as recommended by the manufacturer. Visualisation was done with 3,3′-diaminobenzidine (DAB; Sigma-Aldrich) for 6 min. Stained lung cross-sections were subsequently scanned with a BX51 microscope equipped with the virtual microscopy system dotSlide (Olympus; Tokyo, Japan), and quantitative analysis was performed by measuring the relative area of α-SMA expression normalised to the area of vessel cross-section. For immunofluorescent αSMA and vWF colocalisation, the same protocol for antigen retrieval was used. Then, lung cross-sections were incubated overnight with primary antibodies: anti-vWF antibody (1:200; ab6994; Abcam) overnight at 4°C, and αSMA as described above. A 1:600 dilution of an appropriate FITC-conjugated secondary antibody (111-545-144; Jackson ImmunoResearch) and Cy3-conjugated secondary antibody (111-165-144; Jackson ImmunoResearch) incubated for 30 min at RT were used to visualise immunopositive areas. Images were acquired using a monochromatic AxioCam digital camera and an AxioObserver 22 D1 inverted fluorescent microscope (Carl Zeiss Jena, Oberkochen, Germany).

### 2.9 Lung airness, nuclear, and red blood cell area

Lung airness and relative nuclear area were measured using H&E-stained lung cross-sections scanned with a BX51 microscope equipped with the virtual microscopy system dotSlide (Olympus) and subjected to image segmentation in the Ilastik software according to the appropriate algorithm as previously described (Smeda et al., 2020b). The relative RBC area was measured based on martius yellow, crystal scarlet, and methyl blue (MSB) staining, which stains RBCs yellow. The number of pixels corresponding to RBCs was counted in the ImageJ and is reported as the percentage of lung cross-sections.

### 2.10 Blood count and plasma nitrite/nitrate concentration

Blood was collected in citrate (1:10). Blood count was quantified with a Vet abc animal blood counter (Horiba Medical; Grabels, France), while plasma NO_2_^−^ and NO_3_^−^ concentrations were measured with an ENO-20 NOx Analyzer (Eicom Corp., Kyoto, Japan).

### 2.11 Western blot analysis

Lungs were homogenised in the T-PER buffer (78510; Thermo Fisher Scientific; Waltham, MA, United States) in the presence of protease (11836145001; Roche; Pleasanton, CA, United States) and phosphatase inhibitors (Sigma Aldrich, P0044). Protein concentration was measured with a bicinchoninic acid assay (BCA) kit (23225; Thermo Fisher Scientific). After adding Laemmli Sample Buffer (1610747; Bio-Rad; Hercules, CA, United States) supplemented with 2-mercaptoethanol (1610710; Bio-Rad), samples were heated at 95°C for 5 min and then frozen at −80°C. An equal amount of protein from each sample was loaded and run on a polyacrylamide gel. After protein transfer from the gel to the nitrocellulose membrane, the nitrocellulose membrane was blocked with 5% dry milk and incubated with the primary antibodies directed against the following antigens: p(S1177)eNOS (1:1000; ab195944; Abcam), p(S633)eNOS (1:1000; PA5-64551; Invitrogen), eNOS (1:1000; 610296; BD Transduction Laboratories; Franklin Lakes, NJ, United States), Ang-1 (1:1000; ab8451; Abcam), Ang-2 (1:10000; PA5-27297; Thermo Fisher Scientific), HIF1α (5 μg/ml; ab1; Abcam), VE-CAD (1:1000; sc-6458; Santa Cruz Biotechnology; Dallas, TX, United States), CD31 (1:10000; NBP1-71663H; Novus Biologicals), vascular endothelial growth factor receptor 2 (VEGFR2; 1:1000, ab39256; Abcam), fibronectin (1:2500; MA5-11981; Thermo Fisher Scientific). The horse radish peroxidase (HRP)-conjugated secondary antibodies used were obtained from Santa Cruz Biotechnology (sc-2020, sc-2004, sc-2005) and were used at a 1:5000 concentration. Equal protein loading was confirmed by measuring the total protein loading signal on the lane after transfer with stain free-technique (Rivero-Gutiérrez et al., 2014) using the Bio-Rad ChemiDoc Imager. Densitometric band analysis was performed in the ImageJ software.

### 2.12 Micro RNA analysis

Total RNA from murine plasma was extracted using the miRNeasy Serum/Plasma Advanced Kit (Qiagen; Hilden, Germany) according to the manufacturer’s recommended protocol. Reverse transcription of miRNA was performed using the miRCURY LNA RT Kit (Qiagen), according to the manufacturer’s protocol. MiRNA expression was quantified with real-time quantitative PCR using SYBR Green qPCR Master Mix (EURx; Gdańsk, Poland) with miRCURY LNA miRNA PCR assays (Qiagen) for miRNAs 29a-3p (miR-29a-3p) and 181b-3p (miR-181b-3p), and the U6 small nuclear RNA (snRNA). The miRNA expression levels were quantified using the 2^−ΔΔCT^ method using the U6 snRNA as the endogenous control.

### 2.13 Fourier-transform infrared spectroscopic imaging of pulmonary extracellular matrix remodelling

Paraffin-embedded cross-sections of lungs were cut into 5 µm slices and mounted on IR-transparent windows [calcium fluoride (CaF_2_)] and then dewaxed before FTIR imaging. An Agilent 670-infrared (IR) spectrometer and 620-IR microscope operating in rapid scan mode with a liquid-nitrogen-cooled mercury-cadmium-telluride (MCT) focal-plane array (FPA) detector comprising 16,384 pixels arranged in a 128 × 128 grid format were used to acquire IR images. Transmission spectra were recorded with ×15 Cassegrain objectives collecting 32 scans. Spectra were acquired in the range 3,800 cm^−1^ with a spectral resolution of 4 cm^−1^. FTIR imaging was performed on selected regions of interest (ROI) in the middle of the lung cross-section. Spectral pre-processing and chemometric analysis of IR images were performed at the CytoSpec (v.2.00.01) (CytoSpec, 2021) and MatLab (R2015a) softwares. First, the quality of each pixel-spectrum was evaluated using the sample thickness criteria according to the intensity of the amide I band (1620–1680 cm^−1^). Next, principal component analysis (PCA)-based noise reduction [15 principal components (PCs)] was applied to remove spectral noise. Second, derivative IR spectra were calculated using a Savitzky-Golay algorithm with 13 smoothing points. Then, unsupervised hierarchical cluster analysis (UHCA) was performed in the spectral region from 970 to 1770 cm^−1^. Spectral distances were computed as D-values, and the individual clusters were extracted according to Ward’s algorithm. ROIs were segmented into the regions of atelectasis with low lung airiness, parenchyma with normal airways and thin-walled alveoli, and fibrous/muscular tissue according to a previously reported protocol (Augustyniak et al., 2021). The classification was based on their characteristic spectral profile and confirmed by H&E staining. Mean FTIR spectra of classes were extracted for further analysis. Resonant Mie extended multiplicative signal correction (EMSC) using seven PCs was performed on all spectra (Bassan et al., 2010). Mean second derivative IR spectra were used to calculate integral intensities of selected bands at the OPUS 7.0 software (Bruker Optics; v.7.2.139.1294). Here, a linear baseline was drawn through the peak edges, and the spectrum below that line was integrated over the band’s wavenumber range. Band assignment observed in the fingerprint region of the IR spectrum is summarized in Supplementary Table S1.

### 2.14 Statistical analysis

Statistical analyses were performed in GraphPad Prism v.5.03 (San Diego, CA, United States). We used either parametric tests: unpaired Student’s *t*-test when variances were equal and an unpaired Student’s *t*-test with Welch’s correction when variances differed significantly as well as a two-way analysis of variance (ANOVA) with Bonferroni posthoc test or non-parametric tests: Mann-Whitney U or Kruskal–Wallis with Dunn’s multiple comparison test, depending on the number of groups. The normality of the data distribution was assessed with the Shapiro-Wilk normality test with a variable scale. Data are presented as the median and the interquartile range (IQR) [from lower (25%) to upper (75%) quartile]. Results with *p* ≤ 0.05 were considered significant. Significant outliers identified with Grubbs’ test were excluded from all statistical analyses.

## 3 Results

### 3.1 Systemic endothelial dysfunction and EndMT in the pulmonary circulation of 20-week-old and 40-week-old BALB/c mice

**FIGURE 1:**
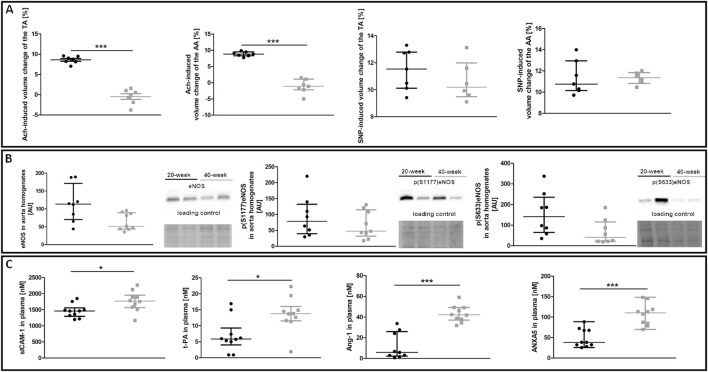
NO-dependent *in vivo* aorta function, eNOS phosphorylation and plasma endothelial dysfunction biomarkers in control untreated 20-week-old and 40-week-old BALB/c mice. Black symbols denote 20-week-old untreated control mice, and grey symbols denote untreated control 40-week-old mice. **(A)** MRI-based *in vivo* NO-dependent function of the aorta. The graphs show changes in the end-diastolic volume of the TA and AA 30 min after ACh or SNP administration (*n* = 6–7). **(B)** The eNOS level and its absolute phosphorylation at S1177 and S633 are shown by representative western blots (*n* = 8–9). **(C)** Changes in selected plasma endothelial dysfunction biomarkers (*n* = 9–10). All data were compared with an unpaired Student’s *t*-test except plasma Ang-1 levels that were compared with a non-parametric Mann-Whitney *U* test. Results are shown as the median and IQR. Key: *p < 0.05; ***p < 0.001.

To characterise age-dependent changes in systemic endothelial function, NO–dependent function of the aorta and plasma nitrite/nitrate concentration were measured in 20-week and 40-week-old control BALB/c mice. While ACh-induced endothelium-dependent vasodilation in the TA and AA of 40-week-old mice was compromised, SNP-induced endothelium-independent vasodilation in the TA and AA was fully preserved (Figure 1A). Systemic endothelial dysfunction in 40-week-old BALB/c mice was associated with a tendency of lower eNOS expression, tendency of lower level of phosphorylated eNOS at S633 in the aorta (Figure 1B), and altered plasma levels of selected systemic endothelial dysfunction biomarkers sICAM-1, t-PA, Ang-1, ANXA5 (Figure 1C). The plasma nitrite concentration of 40-week-old control mice was lower compared to 20-week-old mice (Supplementary Table S2).

**FIGURE 2:**
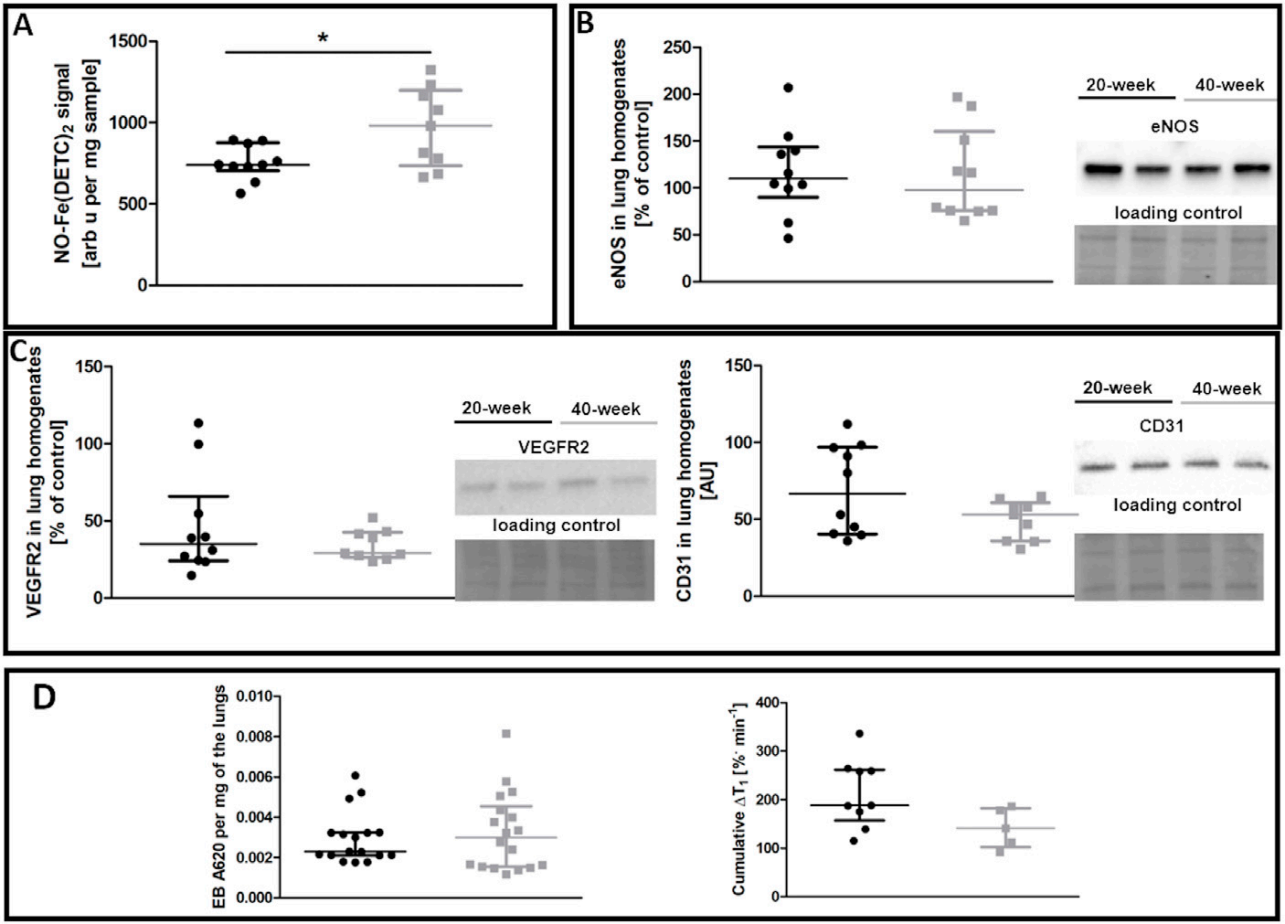
Lung functional parameters of control untreated 20-week-old and 40-week-old BALB/c mice. Black symbols denote 20-week-old untreated control mice, and grey symbols denote untreated control 40-week-old mice. **(A)** iNOS-independent NO production in the lungs (*n* = 9–10). **(B)** eNOS levels in lung homogenates (*n* = 10). **(C)** Levels of other endothelium-specific proteins VEGFR2 and platelet endothelial cell adhesion molecule (CD31) in the lung homogenates are shown by representative Western blots (*n* = 9–10). **(D)** Pulmonary permeability based on the spontaneous EB deposition in the lungs (*n* = 24–26), and the amounts of extravascular liquid (MRI) in the lung parenchyma in response to acute L-NAME treatment (*n* = 5–9). All data were compared using a parametric unpaired Student *t*-test, with Welch’s correction for NO-Fe(DETC)_2_ (reflecting NO production), except for VEGFR2 levels and EB lung deposition which were compared with a non-parametric Mann-Whitney *U* test. The results are shown as the median and IQR. Key: *p < 0.05.

In contrast to the aorta, there was no evidence of compromised NO bioavailability and decreased eNOS levels in the pulmonary circulation of 40-week-old control mice compared to 20-week-old BALB/c mice (Figures 2A,B). Endothelium-specific proteins in the lungs, including VEGFR2 and CD31, were also unchanged (Figure 2C). Pulmonary endothelial permeability based on EB or MRI-based detection of NO-dependent changes in T_1_ was unchanged (Figure 2D).

**FIGURE 3:**
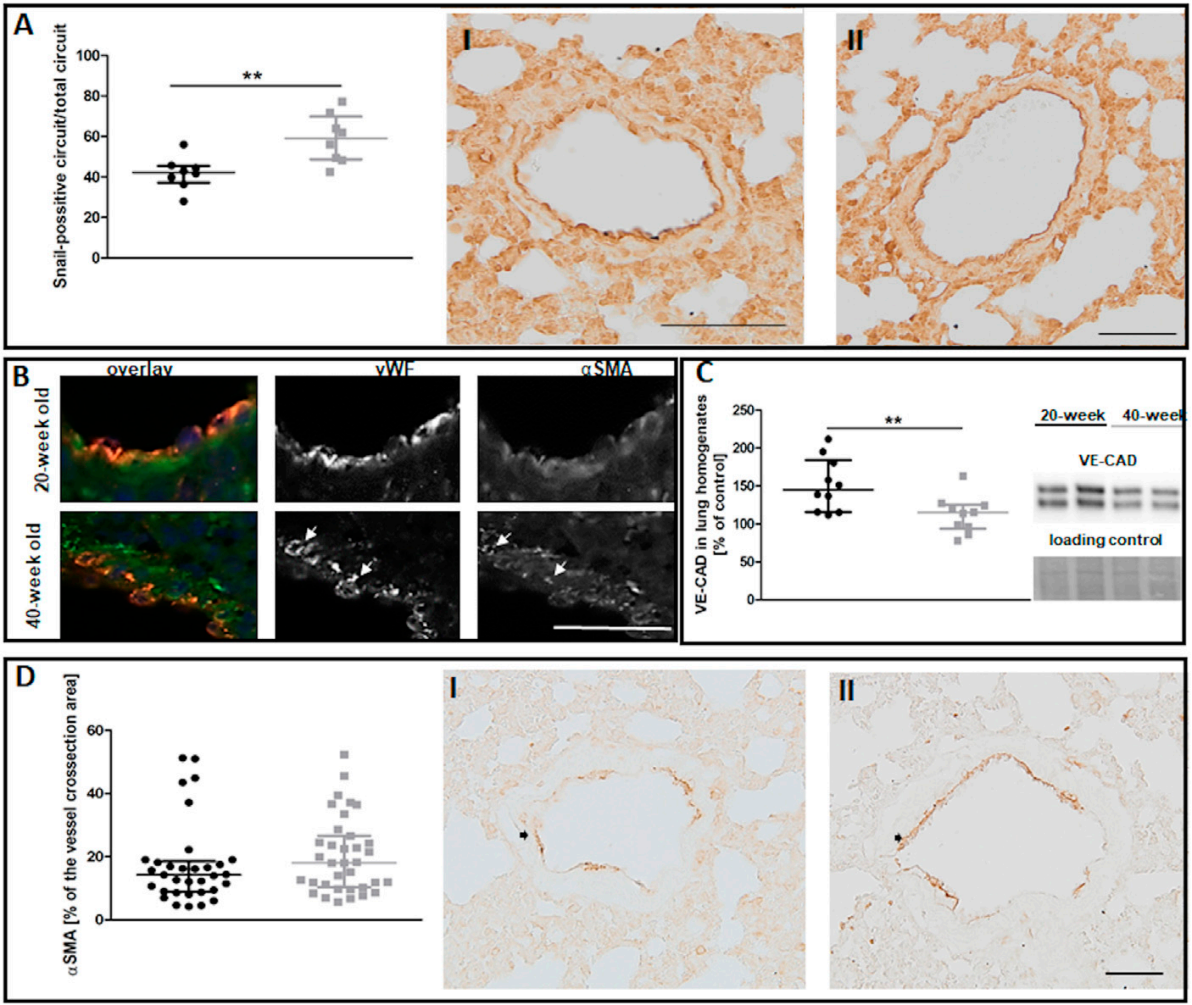
The EndMT in the lungs of untreated 20-week-old and 40-week-old BALB/c mice. Black symbols denote 20-week-old untreated control mice, and grey symbols denote untreated control 40-week-old mice. **(A)** The quantitative analysis of Snail levels in the endothelium of randomly chosen pulmonary vessels. Representative images at ×200 magnification are shown for 20-week-old **(I)** and 40-week-old **(II)** mice (*n* = 8). **(B)** Co-occurrence of vWF and αSMA in lung vascular endothelial cells in untreated control 40-week-old and 20-week-old BALB/c mice is shown at ×400 magnification and indicated by white arrows. **(C)** Quantification of VE-CAD protein levels in lung homogenates of untreated control 20-week-old and 40-week-old mice shown by representative western blots (*n* = 10). **(D)** Quantitative analysis of αSMA levels in randomly chosen pulmonary vessels (*n* = 33–35). Representative images at ×200 magnification are shown for 20-week-old **(I)** and 40-week-old **(II)** mice with the analysed vessels indicated by black arrows. The scale bar in **(A)** represents 100 μm, in **(B)** 50 μm, in **(D)** 50 μm. All data in **(A)** and **(C)** were compared with parametric unpaired Student’s *t*-test. All data in **(D)** were compared with non-parametric Mann-Whitney *U* test. The results are shown as the median and IQR. Key: **p < 0.01.

Despite preserved NO-dependent function in pulmonary circulation, levels of the Snail transcription factor, an early marker of mesenchymal transformation of endothelial cells (Smeda et al., 2018), were increased in the endothelium of selected vessels in 40-week-old control mice compared to 20-week-old mice (Figure 3A). The ongoing EndMT in the lungs of 40-week-old mice was confirmed by the co-occurrence of vWF and mesenchymal marker αSMA (Good et al., 2015) and by lower VE-CAD levels (Figures 3B,C). However, total αSMA levels in the larger lung vessels of 40-week-old mice did not differ from those of 20-week-old mice (Figure 3D).

**FIGURE 4:**
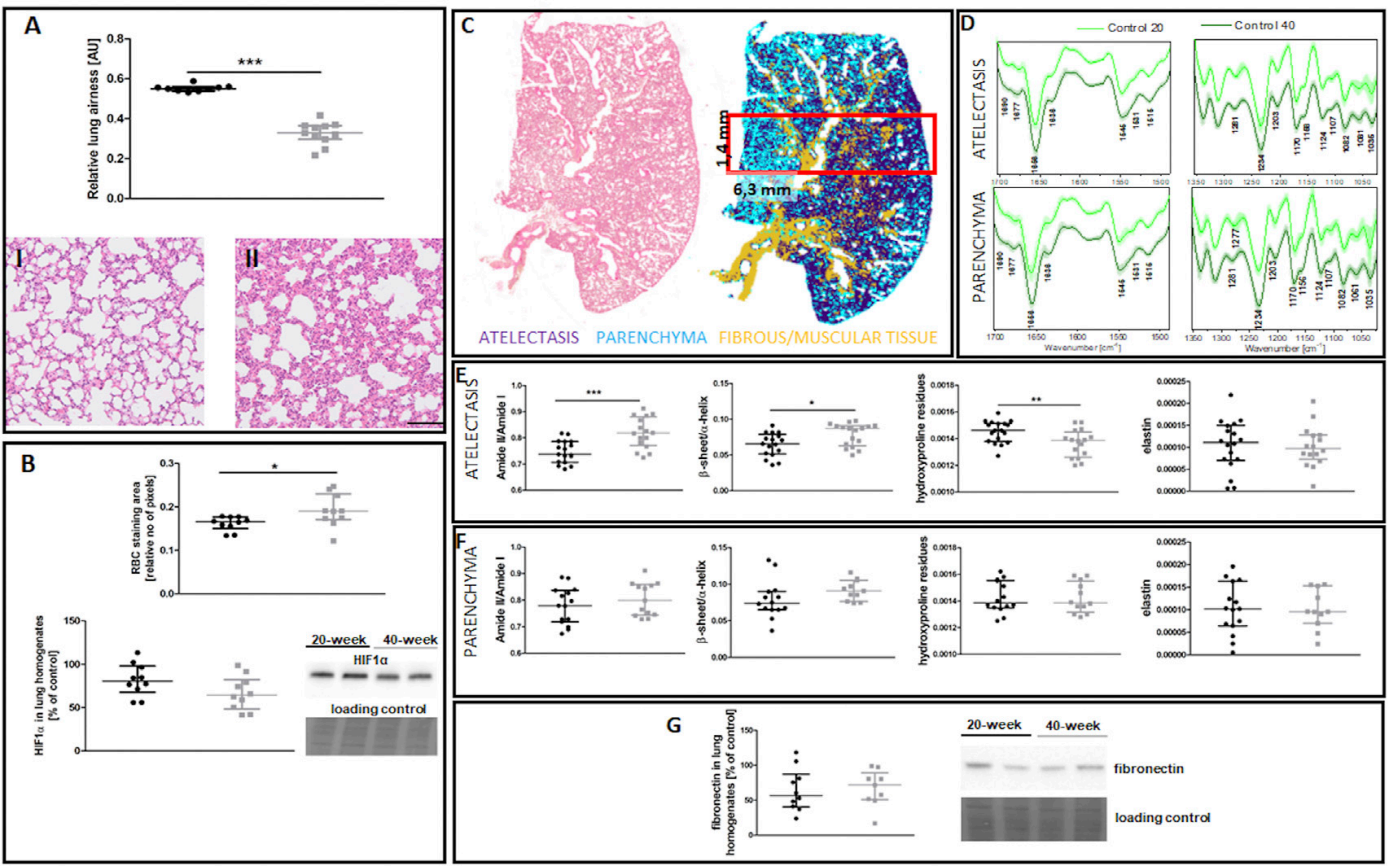
Lung ECM remodelling in untreated control 20-week-old and 40-week-old BALB/c mice. Black symbols denote control untreated 20-week-old mice, and grey symbols denote control untreated 40-week-old mice. **(A)** Relative lung airness (*n* = 10–11). Representative H&E stained sections of lung parenchyma at ×200 magnification for 20-week-old and 40-week-old mice are shown in **(I)** and **(II)**, respectively. **(B)** RBC numbers and HIF1α levels in the lung parenchyma (*n* = 10). A comparison of H&E staining and the false-colour cluster map of the IR image showing differentiation of main morphological structures in lungs is shown in **(C)**, while averaged second derivative FTIR spectra [±standard deviation (SD)] of each group is shown in **(D)**. Semi-quantitative analyses of biomolecules in the atelectasis (*n* = 16–18) **(E)** and **(F)** lung parenchyma (*n* = 10–14), and **(G)** fibronectin levels in lung homogenates (*n* = 10) are shown by a representative western blot. Integration regions: Amide II/I [(1589–1485 cm^−1^)/(1707–1608 cm^−1^)], β-sheet/α-helix [(1640–1623 cm^−1^)/(1670–1640 cm^−1^)], hydroxyproline residues (1187–1140 cm^−1^), and elastin (1070–1040 cm^−1^). The scale bar in **(A)** represents 100 μm. Data are presented as dot plots showing the median and IQR. All data were compared with an unpaired Student’s *t*-test except for β-sheet/α-helix in **(C)** which were compared with a Mann-Whitney *U* test. Key: *p < 0.05; **p < 0.01; ***p < 0.01.

EndMT in the lungs of 40-week-old mice was associated with decreased lung airness (Figure 4A), increased RBC numbers in the lung parenchyma (Figure 4B), and altered biochemical lung tissue composition (Figures 4C–F). The latter finding was based on label-free FTIR imaging analysis that showed an increased Amide II/Amide I ratio and β-sheet/α-helix ratio but decreased hydroxyproline residues in the atelectasis regions (Figures 4E,F). Fibronectin levels were unaltered in the lungs of 40-week-old mice (Figure 4G).

Altogether, these results indicate that 40-week-old mice show age-dependent impairment of endothelial function in the aorta and early EndMT phase in their lungs associated with remodeling in the atelectasis regions.

### 3.2 Effects of 4T1 breast cancer cell intravascular injection on endothelial function in the aorta and endothelial to mesenchymal transition in the pulmonary circulation of 40-week-old and 20-week-old BALB/c mice

To determine whether the observed age-dependent changes affect the response to metastatic cancer cells, we injected i.v. 4T1 breast cancer cells into 20-week-old and 40-week-old BALB/c mice and assessed the endothelial response in the aorta and pulmonary circulation two and seven days post-injection.

#### 3.2.1 Effects of 4T1 breast cancer cell i.v. injection on endothelial phenotype in the aorta and systemic endothelial biomarkers in 40-week-old compared to 20-week-old BALB/c mice

**FIGURE 5:**
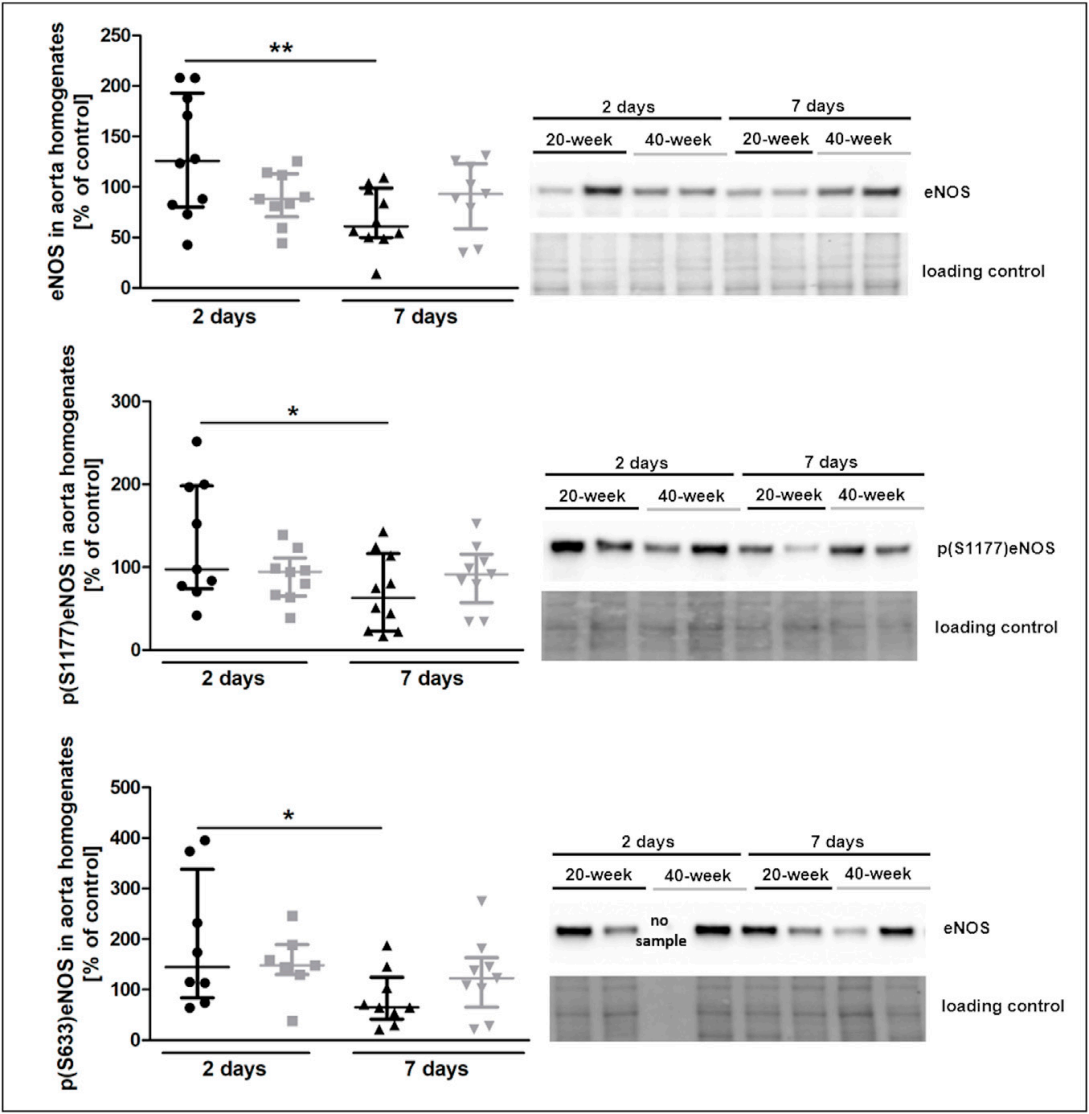
eNOS level and phosphorylation at Ser1177 and Ser633 in the aorta of 4T1 breast cancer cell-injected 20-week-old and 40-week-old BALB/c mice. Black symbols denote 20-week-old mice, and grey symbols denote 40-week-old mice. eNOS level and its absolute phosphorylation at S1177 and S633 in the aorta of 20-week-old and 40-week-old mice two and 7 days post-injection of 4T1 breast cancer cells are shown by representative western blots (*n* = 7–10). The data are shown as the median and IQR and were compared with parametric two-way ANOVA. Key: *p < 0.05; **p < 0.01.

The injection of 4T1 breast cancer cells resulted in increased plasma nitrate concentration in 40-week-old mice seven days after injection (Supplementary Table S3). However, eNOS levels and eNOS phosphorylation at Ser 1177 and Ser 633 were decreased in 20-week mice (Figure 5). Lack of changes in eNOS level and eNOS phosphorylation in the aortas in 40-week-old mice after 4T1 breast cancer cell injection resulted from the initially age-dependent impairment of NO-dependent function in their aortas prior to injection of cancer cells as shown in Figure 1.

**FIGURE 6:**
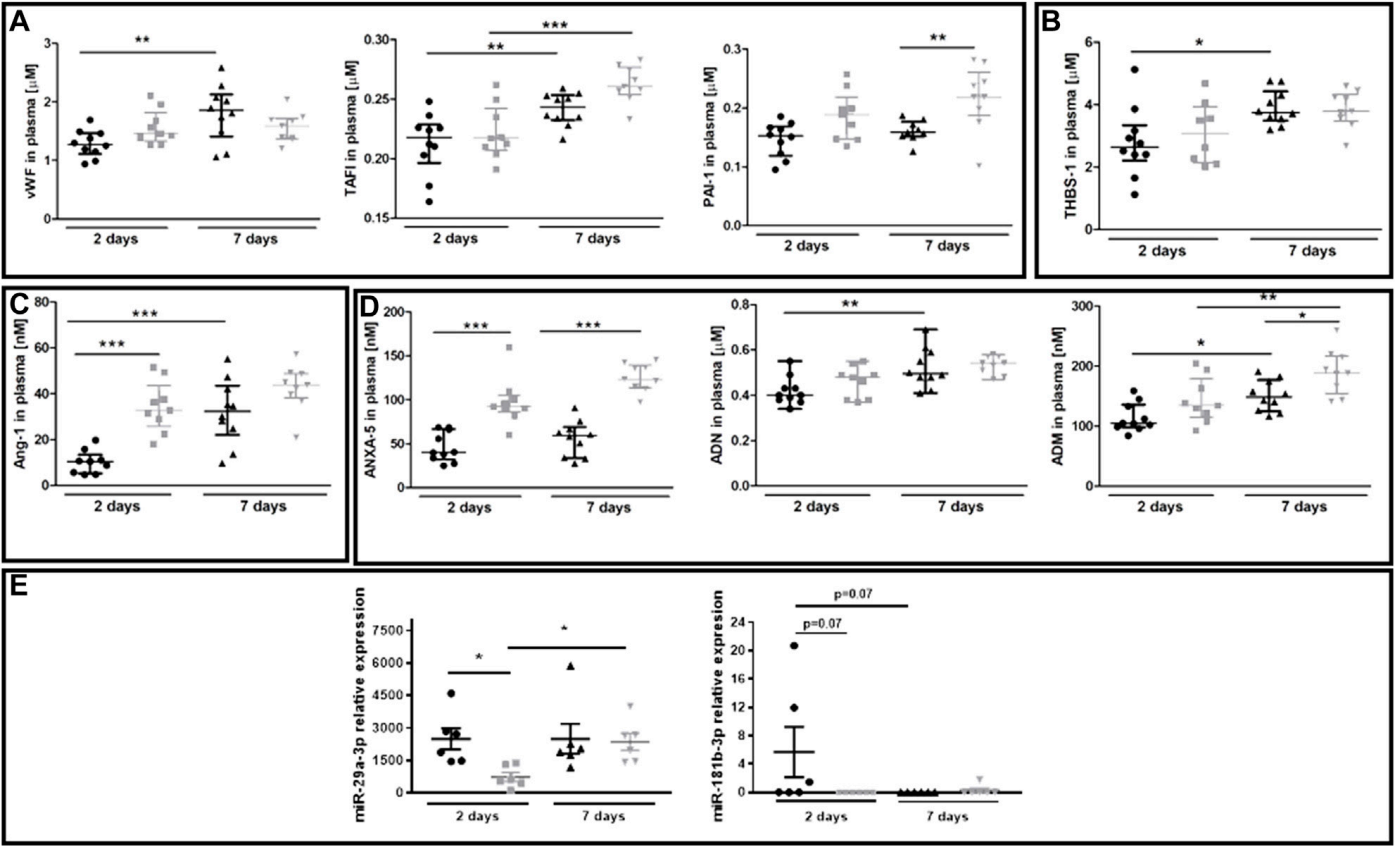
Plasma endothelial dysfunction and EndMT biomarkers in 20-week-old and 40-week-old BALB/c mice after i.v. injecton of 4T1 breast cancer cells. Black symbols denote 20-week-old mice, and grey symbols denote 40-week-old mice. Plasma markers of **(A)** endothelium inflammation and hemostasis (vWF, TAFI, and PAI-1) (*n* = 9–10), **(B)** platelet activation (THBS-1) (*n* = 9–10), **(C)** endothelium permeability (Ang-1) (*n* = 9–10), and **(D)** other biomarkers of endothelial function (ANXA5, ADN, and ADM) are shown two and seven days post-injection of 4T1 breast cancer cells (*n* = 9–10). **(E)** Relative plasma expression levels of potential EndMT markers *miR-29a-3p* and *miR-181b-3p* (*n* = 6). Data are shown as the median and IQR and were compared with parametric two-way ANOVA. Key: *p < 0.05; **p < 0.01; ***p < 0.001.

The plasma concentration of several protein biomarkers of endothelial dysfunction became altered between 40-week-old and 20-week-old BALB/c mice alongside the progression of metastatic disease (Figures 6A–D). These included hemostasis biomarkers vWF, TAFI and PAI-1 (Figure 6A), platelet activation biomarker THBS-1 (Figure 6B), endothelium permeability biomarker Ang-1 (Figure 6C), and other biomarkers regulating angiogenesis or the adhesive endothelium phenotype such as ANXA5, ADN, and ADM (Hinson et al., 2000; Saxena and Sharma, 2010) (Figure 6D). The injection of 4T1 breast cancer cells did not affect plasma concentration of the following biomarkers of endothelial dysfunction: SDC-1, ESM-1, sVCAM-1, sICAM-1, sE-sel, t-PA, sP-sel, sTie-2, Ang-2, and sFLT (results not shown). Finally, we evaluated plasma expression levels of miR-29a-3p and miR-181b-3p which might affect EndMT progression (Srivastava et al., 2019; Green et al., 2021). Plasma expression of miR-29a-3p and miR-181-b-3p was lower in 40-week-old mice than in 20-week-old mice two days post-injection (Figure 6E).

#### 3.2.2 Effects of 4T1 breast cancer cell injection on EndMT in the pulmonary circulation, metastasis, and lung remodelling in 40-week-old compared to 20-week-old BALB/c mice

**FIGURE 7:**
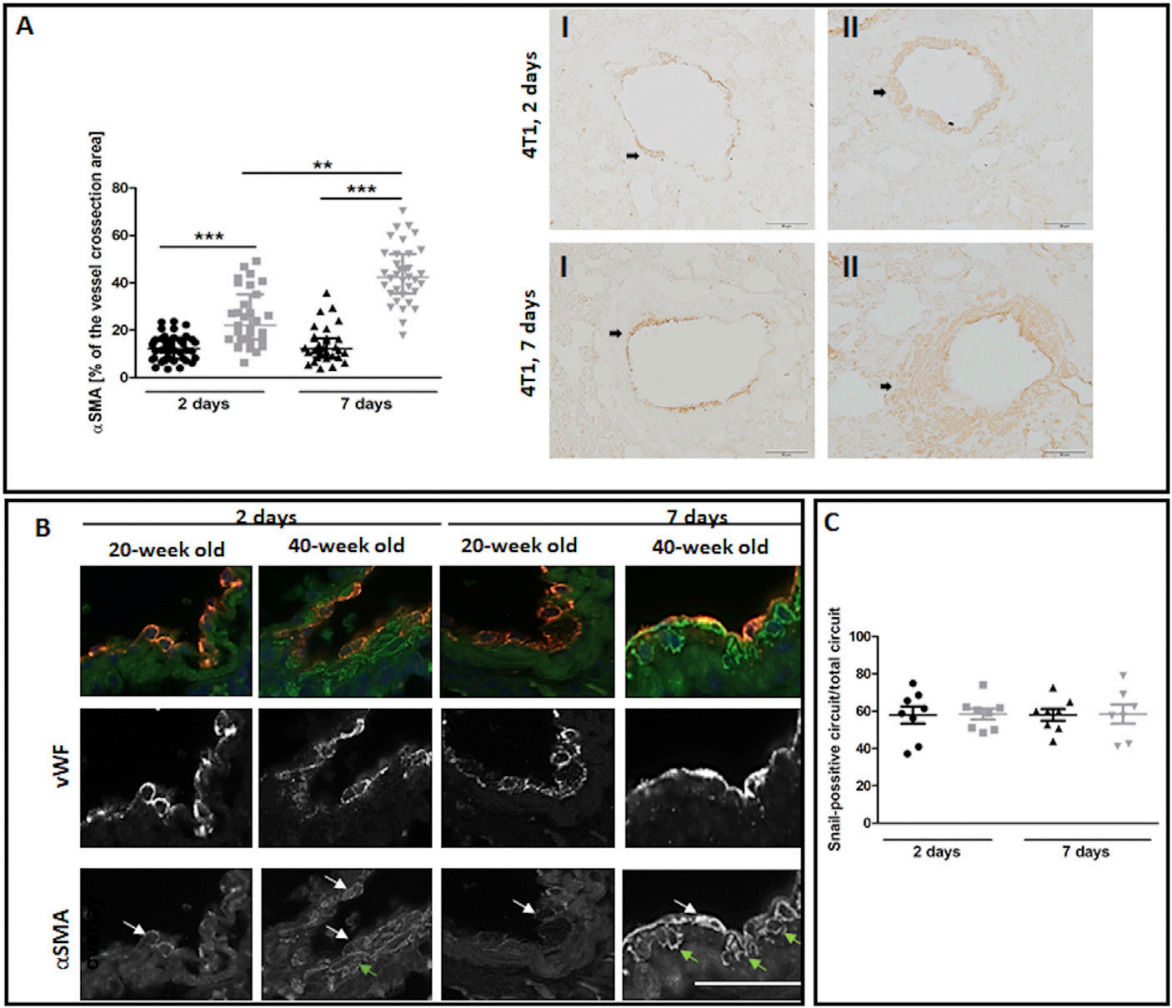
EndMT in lungs of 4T1 breast cancer cell-injected 20-week-old and 40-week-old BALB/c mice. Black symbols denote 20-week-old mice, and grey symbols denote 40-week-old mice. **(A)** Quantitative analysis of αSMA levels in randomly chosen pulmonary vessels (*n* = 31–43). Representative images at ×200 magnification for 20-week-old **(I)** and 40-week-old **(II)** mice at two and seven days post-injection of 4T1 breast cancer cells with αSMA presence indicated by the black arrows. **(B)** Co-occurrence of vWF and αSMA in endothelial cells of lung vessels in 40-week-old mice and 20-week-old mice two and seven days post-injection of 4T1 breast cancer cells is shown at ×400 magnification. The co-occurrence of vWF and αSMA is indicated by white arrows. Green arrows identify cells expressing only αSMA. **(C)** Quantitative analysis of Snail levels in the endothelium of randomly selected pulmonary vessels in mice two and seven days post-injection of 4T1 breast cancer cells (*n* = 7–8). The scale bar in **(A)** and **(B)** represents 50 μm, The data are shown as the median and IQR and were compared with the non-parametric Kruskal–Wallis test **(A)** or two-way ANOVA **(C)**. Key: **p < 0.01; ***p < 0.001.

**FIGURE 8:**
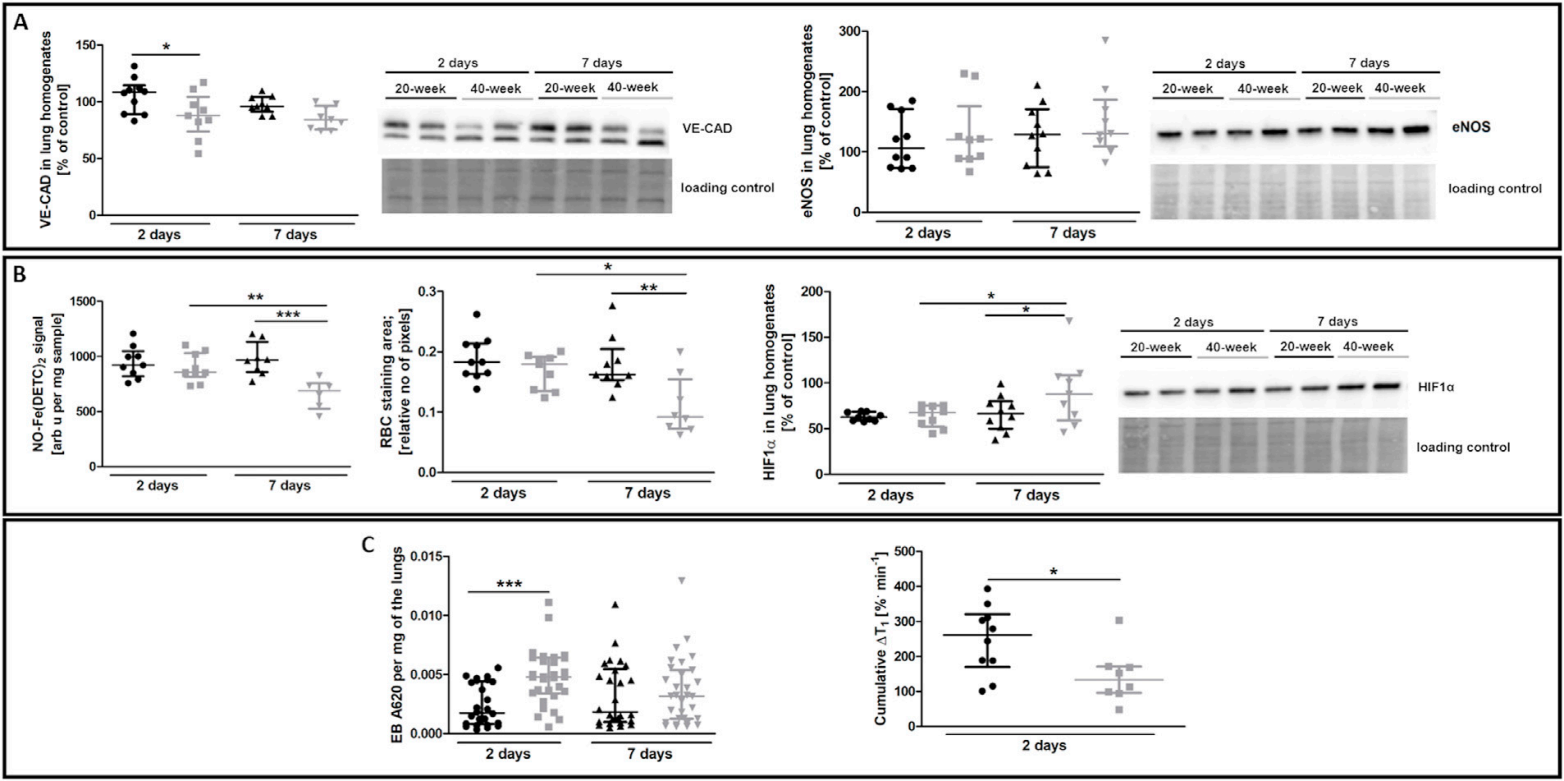
EndMT biomarkers and functional parameters in the lungs of 4T1 breast cancer cell-injected 20-week-old and 40-week-old BALB/c mice. Black symbols denote 20-week-old mice, and grey symbols denote 40-week-old mice. **(A)** VE-CAD and eNOS levels in the lung homogenates of 20-week-old and 40-week-old mice two and seven days post-injection of 4T1 breast cancer cells shown by representative western blots (*n* = 9–10). **(B)** The iNOS-independent NO production (*n* = 6–10), RBC staining area (*n* = 8–10), and HIF1α levels (*n* = 9–10) in the lungs of 20-week-old and 40-week-old mice two and seven days post-injection of 4T1 breast cancer cells are shown by representative western blots. **(C)** Changes in lung permeability indicated by EB dye deposition in the perfused lungs of 20-week-old and 40-week-old mice two and seven days post-injection of 4T1 breast cancer cells (*n* = 26–30) are shown alongside MRI-based quantification of liquid leakage from pulmonary circulation after acute L-NAME treatment (cumulative ΔT_1_) two days post-injection of 4T1 breast cancer cells (*n* = 8–10). Data are shown as the median and IQR and were compared with parametric two-way ANOVA except for eNOS level in lung homogenates **(A)** and EB lung deposition **(C)**, which were compared with a non-parametric Kruskal–Wallis test, and cumulative ΔT_1_, which was compared with an unpaired two-sided Student t test. Key: *p < 0.05; **p < 0.01; ***p < 0.001.

The most noticeable striking difference in response to 4T1 breast cancer cell injection in 40-week-old mice compared to 20-week-old mice was the vessel-specific increase in αSMA levels (Figure 7A). We also found a more pronounced co-occurance of endothelium-specific vWF and αSMA in the pulmonary endothelium of 40-week-old mice compared to 20-week-old mice (Figure 7B). Endothelium-specific Snail levels, known to drive EndMT, were equally high in 20-week-old and 40-week-old mice two and seven days post injection of 4T1 breast cancer cells (Figure 7C) and VE-CAD levels were lower in the lung homogenates of 40-week-old mice compared to 20-week-old mice two days post injection (Figure 8A). While eNOS levels were unaltered (Figure 8A), NO production was significantly compromised in 40-week-old mice compared to 20-week-old mice seven days post-injection (Figure 8B).

RBC numbers found in the lung parenchyma were lower in 40-week-old mice compared to 20-week-old mice suggesting impaired pulmonary capillary perfusion and were consistent with higher HIF1α levels in 40-week-old mice compared to 20-week-old mice after the injection of 4T1 breast cancer cells (Figure 8B). Impaired NO-dependent function was confirmed by MRI-based quantification of liquid leakage from the pulmonary circulation into extracellular space after acute L-NAME treatment (cumulative ΔT_1_; Figure 8C). On the second day post injection, EB-based pulmonary endothelial barrier permeability in the lung parenchyma was also higher in 40-week-old compared to 20-week-old mice (Figure 8C).

**FIGURE 9:**
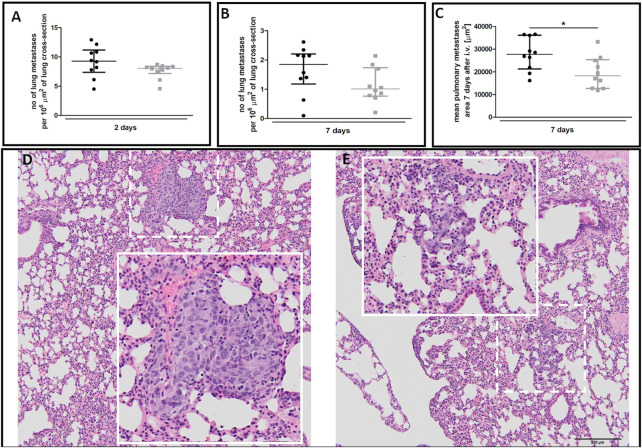
Pulmonary metastasis in 20-week-old and 40-week-old BALB/c mice. Black symbols denote 20-week-old mice, and grey symbols denote 40-week-old mice. The number of pulmonary metastases **(A)** two and **(B)** seven days post-injection of 4T1 breast cancer cells (*n* = 9–10). **(C)** The mean area of pulmonary metastases seven days post-injection of 4T1 breast cancer cells (*n* = 10). The morphology of pulmonary nodules in **(D)** 20-week-old and **(E)** 40-week-old mice seven days post-injection of 4T1 breast cancer cells where the dotted line demarks the area visually enlarged, indicated by the solid line. Data are shown as median and IQR and were compared with a non-parametric Mann-Whitney test **(A,B)** or an unpaired two-sided Student’s *t*-test **(C)**. Key: *p < 0.05.

There was no difference in the number of metastatic cell colonies in 40-week-old and 20-week-old mice on the second (Figure 9A) and seventh (Figure 9B) day post-injection despite rapid EndMT progression in the pulmonary circulation of 40-week-old mice (Figure 7). However, metastatic nodule sizes seven days post-injection were smaller in the lungs of 40-week-old mice (Figure 9C) and their morphology was different with metastatic nodules clearly demarcated in 20-week-old mice (Figure 9D) but diffused in 40-week-old mice (Figure 9E).

**FIGURE 10:**
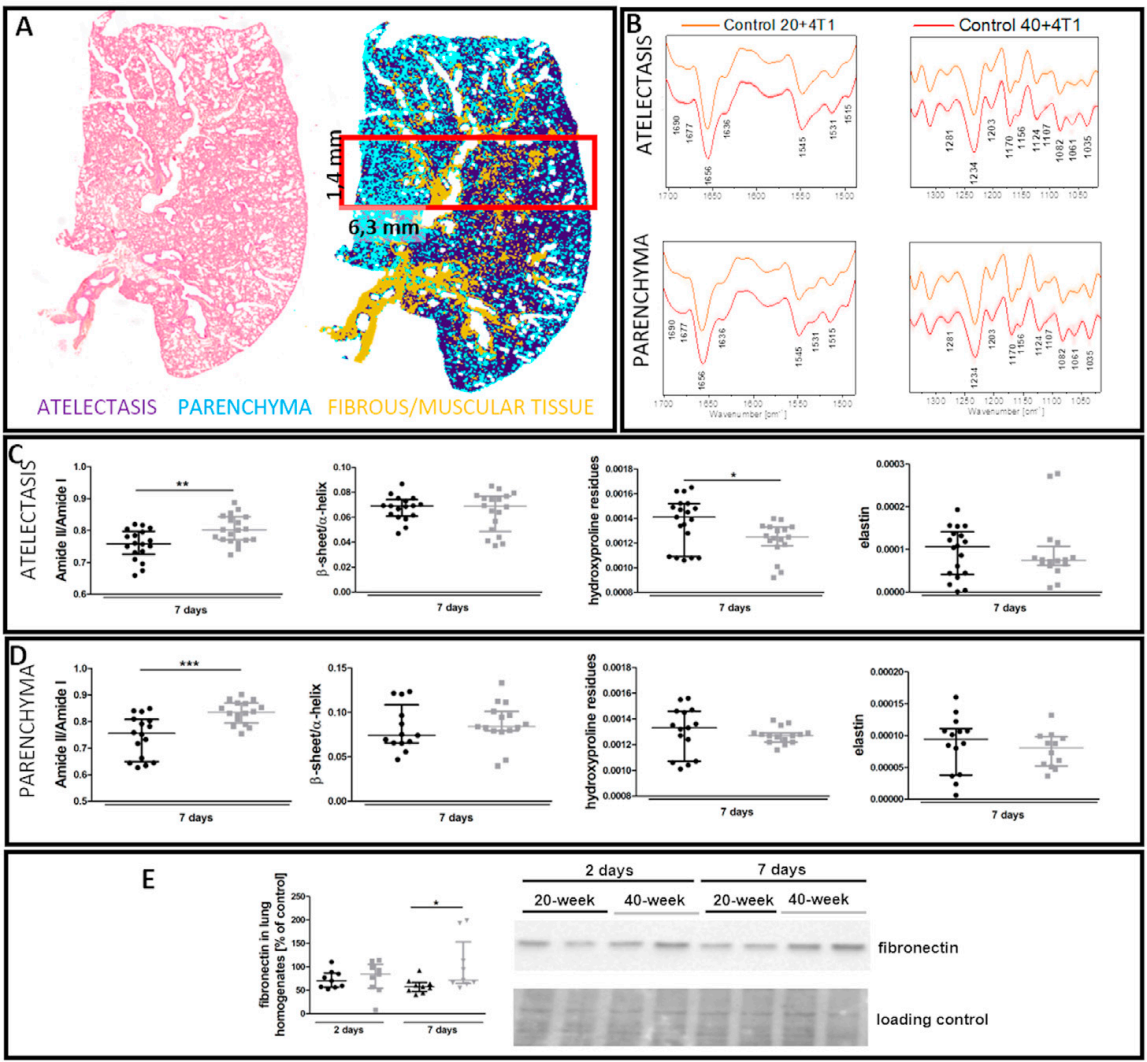
Lung ECM remodelling in 4T1 breast cancer cell-injected 20-week-old and 40-week-old BALB/c mice. Black symbols denote 20-week-old mice, and grey symbols denote 40-week-old mice. **(A)** Comparison of H&E staining and the false-colour cluster map of IR image showing differentiation of main morphological lung structures. **(B)** Averaged second derivative FTIR spectra (±SD) of each group. A semi-quantitative analysis of biomolecules in the **(C)** atelectasis (*n* = 14–20) and **(D)** lung parenchyma (*n* = 12–16), and **(E)** fibronectin levels in the lung homogenates (*n* = 9–10) are shown by representative western blot. Integration regions: Amide II/I [(1589–1485 cm^−1^)/(1707–1608 cm^−1^)], β-sheet/α-helix [(1640–1623 cm^−1^)/(1670–1640 cm^−1^)], hydroxyproline residues (1187–1140 cm^−1^), and elastin (1070–1040 cm^−1^). Data are presented as dot plots with the median and IQR. Amide II/I and β-sheet/α-helix in atelectasis **(C)** and amide II/I, β-sheet/α-helix, hydroxyproline residues, and elastin in the parenchyma **(D)** were compared with an unpaired Student *t*-test. Hydroxyproline residues and elastin in atelectasis were compared with a Mann-Whitney *U* test **(C)** or two-way ANOVA **(E)**. Key: *p < 0.05; **p < 0.01; ***p < 0.01.

FTIR imaging showed that the lungs of 40-week-old mice had increased amide II/I ratios both in the atelectasis and in the parenchyma (Figures 10C,D), while hydroxyproline levels were lower in the atelectasis regions of the lungs of 40-week-old mice (Figures 10C,D). The ECM remodelling process in the lungs of 40-week-old mice induced by the injection of 4T1 breast cancer cells was also reflected by an increase in fibronectin levels in their lungs (Figure 10E).

## 4 Discussion

This study showed that age-dependent impairment of systemic endothelial function in the aorta of 40-week-old mice was associated with preexisting EndMT in the pulmonary endothelium (Figures 1, 3) and pulmonary ECM remodelling (Figure 4). Importantly, we showed that this age-dependent pulmonary endothelial dysfunction phenotype predisposed older mice to rapid EndMT progression in their lungs in response to the presence of metastatic 4T1 breast cancer cells (Figure 7). Our results suggest that rapid EndMT in the lungs of 40-week-old mice in the presence of metastatic 4T1 breast cancer cells might be a major determinant of metastatic breast cancer outcome in older patients because EndMT impairs pulmonary endothelial barrier function (Krenning et al., 2016), which is a critical factor in cancer cell metastasis (Smeda et al., 2020a) and normal organ function (Claesson-Welsh et al., 2021).

Endothelial function deteriorates throughout life due to both ageing and environmental factors (Seals et al., 2011) and is manifested by endothelial barrier dysfunction, vascular hyperpermeability (Oakley and Tharakan, 2014) and progressive NO-dependent vasodilatation impairment (Brandes et al., 2005). Lower eNOS levels or increased oxidant stress impair NO-dependent vasodilation, an important hallmark of age-related endothelial dysfunction. There is evidence suggesting that impaired eNOS-derived NO contributes to EndMT (Kumarswamy et al., 2012) since it limits EndMT by inhibiting VSMC mesenchymal activation (Tsihlis et al., 2011) while decreased eNOS-derived NO facilitates EndMT what, *via* a feedback reinforcement mechanism, further promotes EndMT progression (O’Riordan et al., 2007). We have previously shown that decreased eNOS levels and the resulting NO deficiency in the lungs were associated with EndMT, contributing to the formation of the pre-metastatic niche alongside breast cancer progression in the orthotopic metastatic breast cancer mouse model (Smeda et al., 2018). These events were accompanied by increased vessel-specific levels of transcription factor Snail in the lungs (Smeda et al., 2018), a recognised main driver of EndMT (Cho et al., 2018) that directly downregulates endothelium-specific VE-CAD (Lopez et al., 2009), loosening adherens junctions between adjacent endothelial cells.

This study also found that the endothelium-specific Snail levels were increased in the lungs of 40-week-old BALB/c mice and associated with lower VE-CAD levels, suggesting ongoing age-dependent EndMT in the pulmonary circulation (Figures 3A,C). Indeed, the presence of cells double-stained with vWF and αSMA in the lung vessels of 40-week-old mice (Figure 3B) provides clear evidence (Good et al., 2015) for already ongoing spontaneous age-related EndMT in the pulmonary vessels of 40-week-old compared to 20-week-old mice. Interestingly, age-related EndMT in the lungs of 40-week-old mice was not associated with increased collagen deposition (Supplementary Figures S1–S3) and, therefore lung fibrosis was not activated. On the other hand, EndMT in the lungs of 40-week-old mice was associated with the thickening of the alveolar septa due to deposition of unidentified amorphous material (Supplementary Figure S4). These results indicate that EndMT detected in our experiments represented an early phase response of mesenchymal transition associated with increased production of extracellular matrix but not with robust fibrosis at this stage.

The incidence of EndMT during ageing appears largely unknown (Vidal et al., 2019). Therefore, to our knowledge, this study provides the first evidence for spontaneous age-related EndMT in murine lungs taken from 40-week-old mice. Importantly, the occurrence of EndMT in pulmonary circulation coincided with endothelial dysfunction in the aorta (Figures 1, 3), which are both associated with preserved and impaired NO-dependent function, respectively, underscoring the phenotypic heterogeneity of age-dependent changes in the aorta and pulmonary circulation. Interestingly, we recently observed phenotypic heterogeneity in systemic and pulmonary endothelium in response to diabetes (Fedorowicz et al., 2018). Nevertheless, the co-occurrence of early-stage EndMT in the lungs and impaired endothelium-dependent vasodilation in TA and AA suggest that assessing age-dependent peripheral endothelial dysfunction may be of potential diagnostic and predictive value when evaluating the adverse response of the pulmonary endothelium to an insult resulting in EndMT.

The process of EndMT, driven by cancer cells or other factors (Lopez et al., 2009; Smeda et al., 2018), supports tumour growth and metastasis and resistance to therapeutic treatment (Platel et al., 2019). In this study, i.v. injection of 4T1 breast cancer cells into 20-week-old mice caused EndMT in the pulmonary circulation (Figures 3B, 7B) similarly to the orthotopic metastatic breast cancer mouse model in which we observed several EndMT features in the lungs with pulmonary metastatic progression in young mice (Smeda et al., 2018). However, unlike the lungs of 20-week-old BALB/c mice, those of 40-week-old mice had preexisting EndMT that was substantially accelerated by the presence of 4T1 breast cancer cells (Figures 3, 7). Namely, the preexisting EndMT was associated with decreased lung airness (Figure 4A), increased amide II/I ratio, increased β-sheet/α-helix ratio and decreased lung hydroxyproline content (Figure 4E), indicating altered parenchyma architecture in the atelectasis regions in the lungs of 40-week-old mice (Cissell et al., 2017; Querido et al., 2017; Brauchle et al., 2018). These results support preexisting age-dependent changes in the lung architecture in 40-week-old mice, that were, however, not directly associated with increased fibrosis (Supplementary Figures S1–S3). Interestingly, although injection of 4T1 breast cancer cells increased total collagen deposition both in the vicinity of large vessels and in the lung parenchyma that contains microcirculation (Supplementary Figures S1,S2) but no specifically collagen I and IV (Supplementary Figure S5), there was a clear-cut increase in the thickness of alveoli septae in 40-week-old mice injected with 4T1 breast cancer cells (Supplementary Figure S4) confirming alterations in parenchyma microarchitecture.

Injection of 4T1 breast cancer cells into 40-week-old BALB/c mice also increased fibronectin deposition, a known store for EndMT inducer transforming growth factor β (TGFβ) (Ma et al., 2020) (Figure 10E). Moreover, injection of 4T1 breast cancer cells into 40-week-old but not 20-week-old mice caused a reduction in NO-dependent function, and increased pulmonary endothelial permeability. Altogether, these results suggest that intravascular injection of 4T1 breast cancer cells resulted in more severe impairment of NO-dependent function in 40-week-old BALB/c mice, which could have pro-metastatic effects (Stojak et al., 2018).

Furthermore, the co-occurrence of low RBC numbers in the lungs with increased HIF1α levels in 40-week-old compared to 20-week-old BALB/c mice suggests impaired pulmonary perfusion (Figure 8B) that might also contribute to a hypoxic micro-environment favouring cancer progression (Liu et al., 2015). Surprisingly, despite the number of pro-malignant changes in the lungs, the number of pulmonary metastases did not differ between 40-week-old and 20-week-old mice (Figures 9A,B). Moreover, metastatic nodules in the lungs of 40-week-old mice were smaller (Figure 9C) and had altered morphology (Figures 9D,E) compared to 20-week-old mice. Such an altered cancer cell morphology is consistent with the view that the metastatic tumour cell phenotype depends on local microenvironmental host factors (Anisimov, 2006), which were more unfavourable in the lungs of 40-week-old mice.

## Data Availability

The raw data supporting the conclusions of this article will be made available by the authors, without undue reservation.
