# Erratum: Endothelial-mesenchymal transition induced by metastatic 4T1 breast cancer cells in pulmonary endothelium in aged mice

**DOI:** 10.3389/fmolb.2023.1155174

**Published:** 2023-02-14

**Authors:** Frontiers Production Office

**Affiliations:** Frontiers Media SA, Lausanne, Switzerland

**Keywords:** endothelial-mesenchymal transition, ageing, age-related endothelial dysfunction, breast cancer metastasis, Raman spectroscopy

Due to a production error, Supplementary Figures 1, 2, 3, 4, and 5 were not published.

The publisher apologizes for this mistake. The original version of this article has been updated.

