# Supplementary figures and images for: Endothelial-mesenchymal transition induced by metastatic 4T1 breast cancer cells in pulmonary endothelium in aged mice
Source: Front Mol Biosci. 2022 Nov 24;9:1050112. doi: 10.3389/fmolb.2022.1050112 (PMC9731229; doi:10.3389/fmolb.2022.1050112)

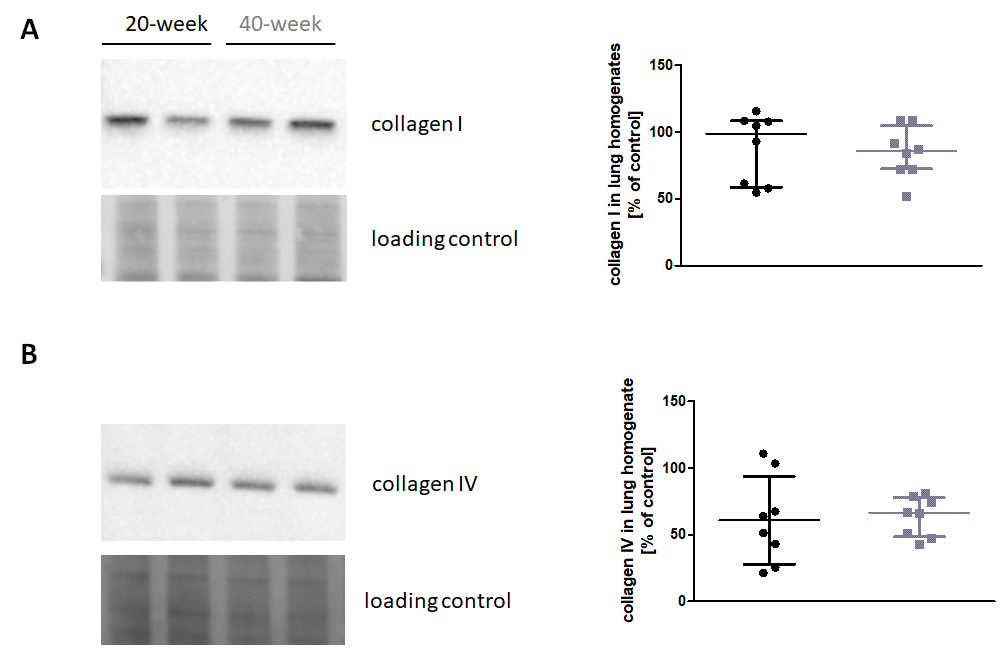

Supplement: Supplementary file 1 [file Image3.TIF]

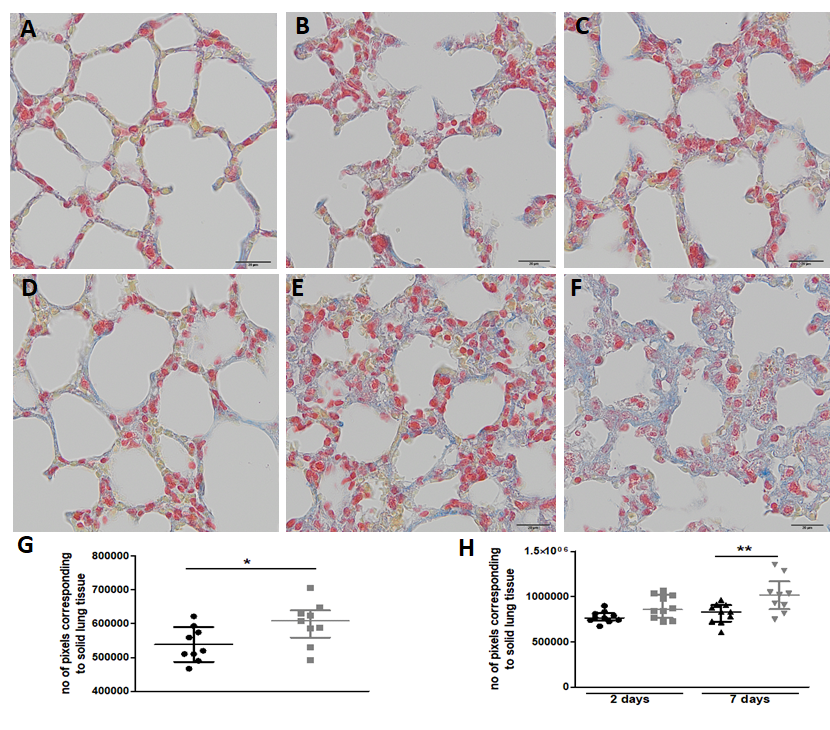

Supplement: Supplementary file 2 [file Image4.TIF]

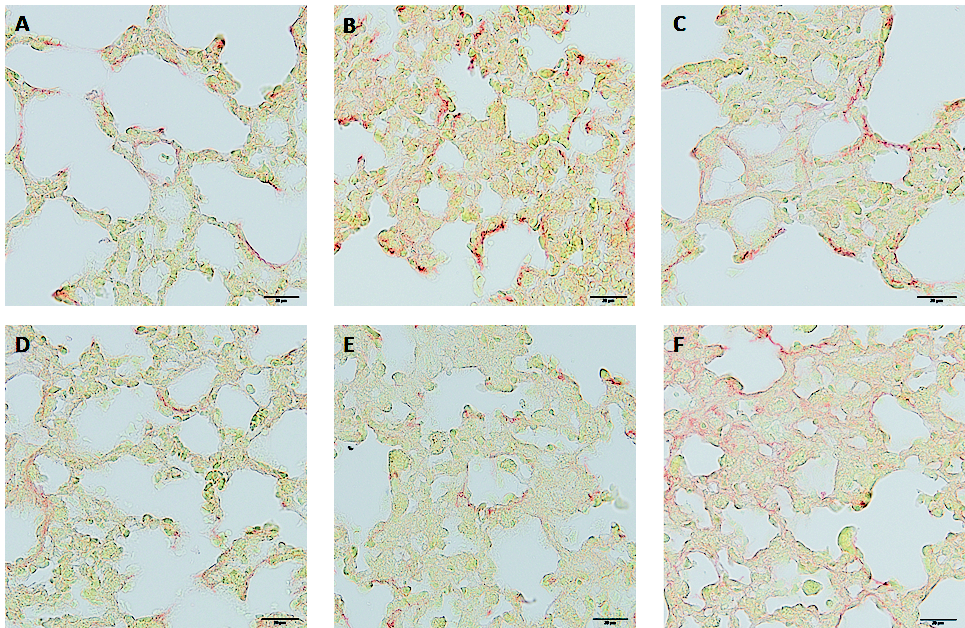

Supplement: Supplementary file 3 [file Image2.TIF]

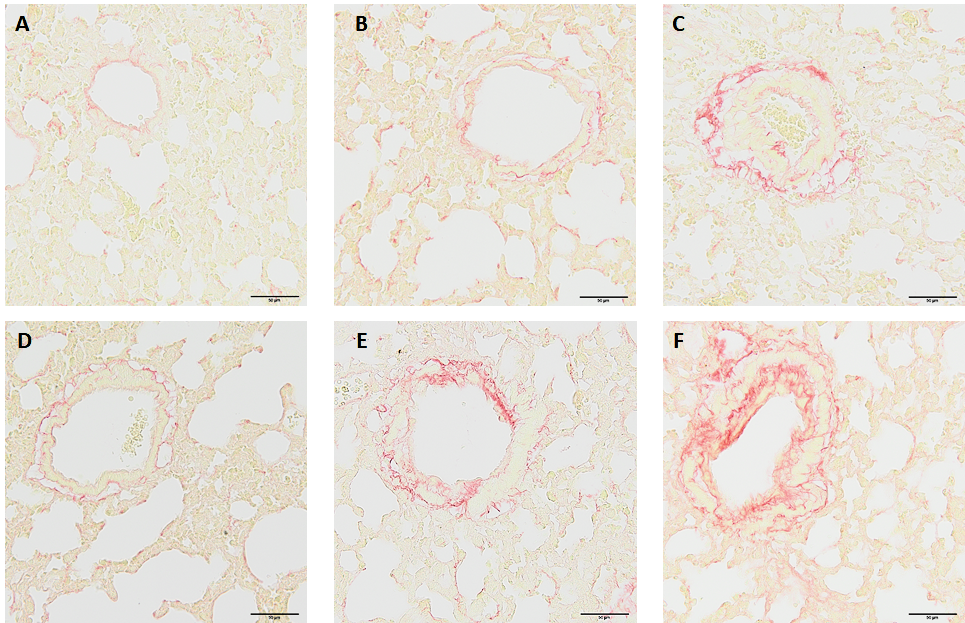

Supplement: Supplementary file 4 [file Image1.TIF]

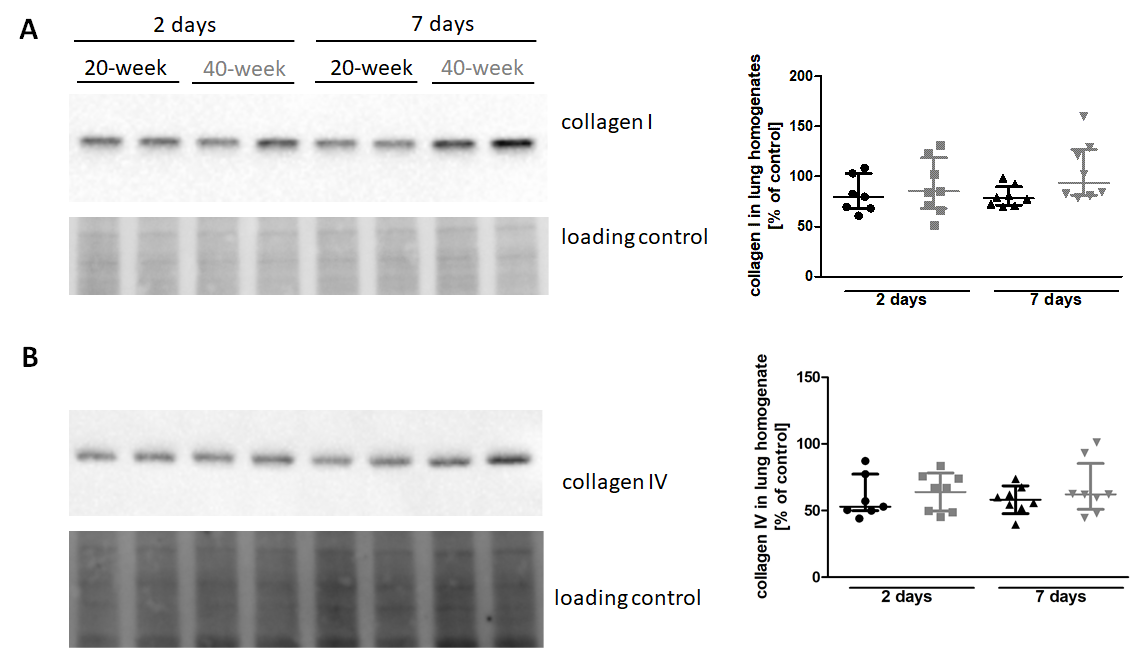

Supplement: Supplementary file 6 [file Image5.TIF]
