# Supplementary material for: Endothelial-mesenchymal transition induced by metastatic 4T1 breast cancer cells in pulmonary endothelium in aged mice
Source: Front Mol Biosci. 2022 Nov 24;9:1050112. doi: 10.3389/fmolb.2022.1050112 (PMC9731229; doi:10.3389/fmolb.2022.1050112)
Supplement: Supplementary file 5 [file DataSheet2.docx]

**Supplementary Figure S1. Qualitative assessment of total collagen content in pulmonary interstitial large vessels of BALB/c mice intravenously injected with 4T1 breast cancer cells.** Lung cross-sections of untreated 20-week-old mice (A) and 4T1 breast cancer cell-injected 20-week-old mice 2 (B) and 7 days (C) post injection as well as lung cross-sections of untreated 40-week-old mice (D) and 4T1 breast cancer cell-injected 40-week-old mice 2 (E) and 7 days (F) post injection were stained with Picrosirius red (PSR) to visualise total collagen content (red), scanned, and subjected to qualitative assessment. The representative images of large vessel collagen deposition in untreated and 4T1-breast cancer cell injected BALB/c mice of both age groups are shown in (A)-(F). Scale bar corresponds to 50 μm (magnification 400x).

**Supplementary Figure S2. Qualitative assessment of total collagen content in lung parenchyma containing pulmonary microcirculation of BALB/c mice intravenously injected with 4T1 breast cancer cells.** Lung cross-sections of untreated 20-week-old mice (A) and 4T1 breast cancer cell-injected 20-week-old mice 2 (B) and 7 days (C) post injection as well as lung cross-sections of untreated 40-week-old mice (D) and 4T1 breast cancer cell-injected 40-week-old mice 2 (E) and 7 days (F) post injection were stained with Picrosirius red (PSR) to visualise total collagen content (red), scanned, and subjected to qualitative assessment. The representative images of lung parenchyma collagen deposition in untreated and 4T1-breast cancer cell injected BALB/c mice of both age groups are shown in (A)-(F). Scale bar corresponds to 20 μm (magnification 400x).

**Supplementary Figure S3. (A) collagen I and (B) collagen IV in the lung homogenates of 20-week-old (black in the graph) and 40-week-old (grey in the graph) mice**. The results are shown as median ± IQR and were analysed with unpaired two-sided Student T test (n=8).

**Supplementary Figure S4. Amount of solid lung tissue in the lung parenchyma of 20-week-old and 40-week-old mice.** The (A)-(F) show the representative pictures of lung parenchyma with exclusion of large vessels and bronchi. The lung cross-sections were stained with MSB staining (*maritius yellow* for visualisation of RBC, *scarlet red* for visualisation of fibrin and cell nuclei, and *methyl blue* for visualisation of cell cytoplasm, extracellular matrix, and collagen). The lung parenchyma of untreated 20-week-old mice is shown in (A) and the lung parenchyma of 20-week-old mice injected with 4T1 breast cancer cells 2 and 7 days after injection is shown in (B) and (C), respectively. The lung parenchyma of untreated 40-week-old mice is shown in (D) and the parenchyma of 40-week-old mice injected with 4T1 breast cancer cells 2 and 7 days after injection is shown in (E) and (F), respectively. The (G) and (H) show quantitative analysis of the number of pixels corresponding to the amount of solid lung tissue excluding air spaces, RBCs, and cell nuclei in untreated 20-week-old mice (black) and 40-week-old mice (grey) (G) and in 20-week-old mice (black) and 40-week-old mice (grey) injected with 4T1 breast cancer cells (H). Ten random pictures of the lung parenchyma (excluding bronchi and large vessels) were taken for each mouse and analysed in Image J to count the number of pixels corresponding to ECM and cell cytoplasm with exclusion of cell nuclei and RBCs. The results of ten pictures were averaged for each mouse. Statistical analysis was performed with either unpaired two-sided Student T-test and the results are shown as the median and IQR (G) or with two-way ANOVA and the results are shown as median ± IQR (H). Scale bar corresponds to 20 μm (magnification 400x).

**Supplementary Figure S5. (A) collagen I and (B) collagen IV in the lung homogenates of 20-week-old (black on the graph) and 40-week-old (grey in the graph) mice 2 and 7 days after injection of 4T1 breast cancer cells**. The results are shown as median ± IQR and were analysed with two-way ANOVA (n=8).
