# Supplementary material for: Endothelial-mesenchymal transition induced by metastatic 4T1 breast cancer cells in pulmonary endothelium in aged mice
Source: Front Mol Biosci. 2022 Nov 24;9:1050112. doi: 10.3389/fmolb.2022.1050112 (PMC9731229; doi:10.3389/fmolb.2022.1050112)
Supplement: Supplementary file 7 [file DataSheet1.docx]

Supplementary Material

# Supplementary Tables

| **Vibration modes** | **Position [cm^-1^]** | **Assignment** |
| --- | --- | --- |
| ν(C=O), amide I: β-turns, β-sheets | 1690-1670 | Proteins |
| ν(C=O), amide I: α-helices | 1656 | Proteins |
| ν(C=O); amide I, parallel β-sheets | 1636 | Proteins |
| δ(NH); amide II of proteins | 1545 | Proteins |
| δ(NH); amide II of collagen scaffolds | 1531 | Proteins |
| in-plane δ(CH) of phenyl ring | 1515 | Tyrosine residues |
| ν(C-N)/δ(N-H)/ν(CH_3_-C): amide III | 1310-1202 | Fibrous proteins |
| ν(C-OH) | 1170 | Hydroxyproline residues |
| δ(C-O-H), δ(C-O-C) | 1061 | Elastin |
| ν(C-O), δ(C-O-H), δ(C-O-C) | 1124, 1082, 1035 | Collagen-associated carbohydrate moieties |

**Supplementary Table S1.** **Assignment of the major bands of FTIR spectra**. An assignment of the major bands of FTIR spectra is depicted in Figures 4 and 10.

|  |  | | **20-week old** | **40-week old** |
| --- | --- | --- | --- | --- |
| **plasma nitrite [μM]** |  | 0.0090 (0.0035; 0.0610) | | 0.0 (0.0; 0.00025)* |
| **plasma nitrate [μM]** |  | 8.675 (5.279; 10.77) | | 4.885 (3.580; 7.947) |
| **lung weight [% of BW]** |  | 0.6055 (0.5791; 0.6388) | | 0.6531 (0.5733; 0.7331) |
| **lung relative nuclear area [AU]** |  | 0.3042 (0.2895; 0.3236) | | 0.3284 (0.2968; 0.3641) |
| **WBC [K^.^μl^-1^]** |  | 2.650 (2.300; 3.075) | | 1.900 (1.200; 2.900) |
| **MON [%]** |  | 9.200 (7.125; 10.780) | | 9.800 (8.800; 11.500) |
| **GRA [%]** |  | 15.90 (12.28; 20.20) | | 14.80 (9.60; 18.70) |
| **LYM [%]** |  | 73.95 (69.93; 80.68) | | 77.40 (70.20; 80.20) |
| **PLT [K^.^μl^-1^]** |  | 792.0 (701.3; 947.3) | | 831.0 (757.5; 913.0) |
| **MPV [fl]** |  | 4.850 (4.700; 4.925) | | 4.800 (4.675; 4.900) |

**Supplementary Table S2.** **Plasma nitrite/nitrate, basic blood and lung parameters** **in untreated control 20-week and 40-week old BALB/c mice.** WBC (white blood cells), MON (monocytes), GRA (granulocytes), LYM (lymphocytes), PLT (platelets), MPV (mean platelet volume). The data are presented as the median and IQR and were analysed with parametric unpaired two-sided Student t-test (plasma nitrate, lung weight, lung relative nuclear area, MON, GRA, LYM, PLT, MPV) or non-parametric Mann-Whitney test (plasma nitrite, WBC). The symbol * denotes statistical significance at P<0.05 between untreated control 20-week and 40-week old mice.

|  | **20-week old, 2 days** | **40-week old, 2 days** | **20-week old, 7 days** | **40-week old, 7 days** |
| --- | --- | --- | --- | --- |
| **plasma nitrite [μM]** | 0.0000 (0.0000; 0.0035) | 0.0000 (0.0000; 0.0135) | 0.0000 (0.0000; 0.03225) | 0.0000 (0.0000; 0.0000) |
| **plasma nitrate [μM]** | 8.1730 (7.1430; 12.8800) | 6.6890 (4.8310; 9.4120) | 6.1940 (3.2740; 9.1670) | 19.3400 (6.9130; 19.3400)*, ^##^ |
| **lung weight [% of BW]** | 0.6818 (0.6425; 0.7225) | 0.6515 (0.5185; 0.8137) | 0.7143 (0.6755; 0.7754) | 0.9828 (0.8186; 1.2650)***,^###^ |
| **lung relative nuclear area [AU]** | 0.2879 (0.2786; 0.3304) | 0.2771 (0.2633; 0.3036) | 0.3103 (0.2864; 0.3918) | 0.3534 (0.3174; 0.3857)** |
| **WBC [K^.^μl^-1^]** | 2.1500 (1.7500; 2.7500) | 3.8000 (2.8500; 4.4500) | 4.6500 (3.3500; 6.2500)^aa^ | 5.8000 (4.6500; 6.7250) |
| **MON [%]** | 8.5500 (5.9000; 9.1750) | 8.6000 (6.6000; 10.9500) | 10.7500 (9.9750; 12.0500)^a^ | 13.4000 (10.3000; 14.0000)** |
| **GRA [%]** | 13.8000 (10.9500; 17.2000) | 14.1000 (11.0500; 16.4500) | 20.5500 (18.8000; 25.1000)^aaa^ | 16.7000 (14.4000; 19.7000)^#^ |
| **LYM [%]** | 77.7500 (73.1500; 82.9000) | 76.0000 (72.7500; 79.1500) | 68.6500 (63.2300; 70.9000)^aaa^ | 69.9000 (67.0000; 75.4500)* |
| **PLT [K^.^μl^-1^]** | 735.5 (603.0; 877.0) | 634.0 (422.5; 673.5) | 769.5 (687.8; 819.0) | 608.0 (494.5; 749.0)* |
| **MPV [fl]** | 4.700 (4.675; 4.800) | 4.700 (4.625; 4.800) | 4.850 (4.800; 5.025) | 4.900 (4.850; 5.300)* |

**Supplementary Table S3.** **Plasma nitrite/nitrate, basic blood and lung parameters in 4T1 breast cancer cell-injected 20- and 40-week old BALB/c mice**. WBC (white blood cells), MON (monocytes), GRA (granulocytes), LYM (lymphocytes), PLT (platelets), MPV (mean platelet volume). The data are presented as the median and IQR and were analysed with parametric two-way ANOVA (plasma nitrate, lung weight, MON, GRA, LYM) or non-parametric Mann-Whitney test (plasma nitrite, lung relative nuclear area, WBC, PLT, MPV). The symbols *, ** and *** denote statistical significance at P<0.05, P<0.01, and P<0.001, respectively, between 40-week old mice 2 and 7 days after i.v.; the symbols ^a^, ^aa^ and ^aaa^ denote statistical significance at P<0.05, P<0.01, and P<0.001, respectively, between 20-week old mice 2 and 7 days after i.v.; the symbols ^#^, ^##^ and ^###^ denote statistical significance at P<0.05, P<0.01, and P<0.001, respectively, between 20-week and 40-week old mice 7 days after i.v.
